# Barriers and facilitators influencing the sustainment of health behaviour interventions in schools and childcare services: a systematic review

**DOI:** 10.1186/s13012-021-01134-y

**Published:** 2021-06-12

**Authors:** Adam Shoesmith, Alix Hall, Luke Wolfenden, Rachel C. Shelton, Byron J. Powell, Hannah Brown, Sam McCrabb, Rachel Sutherland, Serene Yoong, Cassandra Lane, Debbie Booth, Nicole Nathan

**Affiliations:** 1 School of Medicine and Public Health, The University of Newcastle, University Drive, Callaghan, 2308 NSW Australia; 2 Hunter New England Population Health, Hunter New England Local Health District, Locked Bag No. 10, Wallsend, NSW 2287 Australia; 3 Priority Research Centre for Health Behaviour, The University of Newcastle, University Drive, Callaghan, 2308 NSW Australia; 4 Hunter Medical Research Institute, 1/Kookaburra Circuit, New Lambton Heights, 2305 NSW Australia; 5 Department of Sociomedical Sciences, Mailman School of Public Health, Columbia University, New York, NY 10032 USA; 6 Brown School and School of Medicine, Washington University in St. Louis, One Brookings Drive, Campus Box 1196, St. Louis, MO 63130 USA; 7 University Library, Academic Division, University of Newcastle, University Drive, Callaghan, NSW 2308 Australia

**Keywords:** Sustainability, Sustainment, Schools, Childcare, Interventions, Guidelines, Barriers, Facilitators, Factors

## Abstract

**Background:**

Sustainment has been defined as the sustained use or delivery of an intervention in practice following cessation of external implementation support. This review aimed to identify and synthesise factors (barriers and facilitators) that influence the sustainment of interventions (policies, practices, or programmes) in schools and childcare services that address the leading risk factors of chronic disease.

**Methods:**

Seven electronic databases and relevant reference lists were searched for articles, of any design, published in English, from inception to March 2020. Articles were included if they qualitatively and/or quantitatively reported on school or childcare stakeholders’ (including teachers, principals, administrators, or managers) perceived barriers or facilitators to the sustainment of interventions addressing poor diet/nutrition, physical inactivity, obesity, tobacco smoking, or harmful alcohol use. Two independent reviewers screened texts, and extracted and coded data guided by the Integrated Sustainability Framework, an existing multi-level sustainability-specific framework that assesses factors of sustainment.

**Results:**

Of the 13,158 articles identified, 31 articles met the inclusion criteria (8 quantitative, 12 qualitative, 10 mixed-methods, and 1 summary article). Overall, 29 articles were undertaken in schools (elementary *n*=17, middle *n*=3, secondary *n*=4, or a combination *n*=5) and two in childcare settings. The main health behaviours targeted included physical activity (*n*=9), diet (*n*=3), both diet and physical activity (*n*=15), and smoking (*n*=4), either independently (*n*=1) or combined with other health behaviours (*n*=3). Findings suggest that the majority of the 59 barriers and 74 facilitators identified to impact on intervention sustainment were similar across school and childcare settings. Factors predominantly relating to the ‘inner contextual factors’ of the organisation including: availability of facilities or equipment, continued executive or leadership support present, and team cohesion, support, or teamwork were perceived by stakeholders as influential to intervention sustainment.

**Conclusions:**

Identifying strategies to improve the sustainment of health behaviour interventions in these settings requires a comprehensive understanding of factors that may impede or promote their ongoing delivery. This review identified multi-level factors that can be addressed by strategies to improve the sustainment of such interventions, and suggests how future research might address gaps in the evidence base.

**Trial registration:**

This review was prospectively registered on PROSPERO: CRD42020127869, Jan. 2020.

**Supplementary Information:**

The online version contains supplementary material available at 10.1186/s13012-021-01134-y.

Contributions to the literature

- Previous studies have documented challenges related to the sustainability of intervention delivery across a range of clinical and community-based settings. This review is the first to identify and synthesise barriers and facilitators influencing sustainment of health behaviour interventions employed in school and childcare settings using a sustainability-specific framework.
- Barriers and facilitators predominantly relating to ‘inner contextual factors’ of the organisation including: availability of facilities or equipment, continued executive or leadership support present, and team cohesion, support, or teamwork were perceived by stakeholders as most influential to intervention sustainment.
- These findings contribute to the existing literature regarding the sustainment of evidence-based interventions, and help inform the future development and empirical testing of strategies to address barriers and facilitate the sustainment of health behaviour interventions, supporting positive, long-term health outcomes in children.

## Background

Globally, chronic diseases are the leading cause of morbidity and mortality, responsible for 70% of all deaths [1, 2]. Poor diet, physical inactivity, obesity, tobacco smoking, and harmful alcohol use are the primary behavioural risk factors for chronic disease development across the life course [3, 4]. As many of these risk factors develop in childhood and track into adulthood, efforts to address these health behaviours in the early, developmental years of life is warranted [3]. Educational settings that target younger age groups (i.e., childcare services and schools) are widely recommended as ideal locations for health behaviour interventions (policies, practices, and programmes) as they enable the targeting of key risk factors, provide reach to a large segment of children, and offer opportunities for continuous and intensive contact with children for prolonged periods [5]. Systematic review evidence also suggests that many effective interventions targeting these risk factors in these settings now exist [6–11].

To achieve health benefits at the population level, effective interventions targeting these risk factors need to be implemented in an ongoing manner in these settings [12, 13]. Recent Cochrane systematic reviews show promising effects of multi-component interventions and implementation strategies delivered in schools and childcare services [14, 15]; however, few have described the long-term sustainment of intervention delivery [12, 13]. Sustainment has been defined as ‘the sustained use or delivery of an intervention in practice following cessation of external implementation support’ [16, 17]. Findings of systematic reviews suggest that intervention sustainment is a considerable challenge, fraught with challenges in evaluation and real-world logistics [12, 18]. For example, a recent review of the sustainment of health behaviour interventions in schools found that of the 18 included interventions, none were sustained in their entirety following the cessation of external implementation support [19]. Additionally, impediments to intervention sustainment in childcare services identified by Ward et al. highlight the need to investigate avenues to increase long-term sustainment within this setting [20]. In light of the well-documented difficulties to intervention sustainment [18], an understanding of the barriers and facilitators that influence intervention sustainment across settings is needed to inform the development of future sustainability strategies.

Two reviews have reported on the factors influencing sustainment of health behaviour interventions in schools [19, 21]. A 2019 review by Cassar et al. [21] aimed to identify factors associated with the adoption, implementation, and sustainability of school-based physical activity interventions in real-world settings. Seven interventions conducted in high-income countries were identified, from which 63 factors (33 facilitators and 30 barriers) were found to influence sustainment of physical activity interventions in schools. When mapped to the five domains of Durlak and DuPre’s implementation model [22], these included community-level factors (e.g., funding), provider characteristics (e.g., the perceived need and benefit of the innovation), characteristics of the innovation (e.g., the compatibility of the intervention with the setting), factors relevant to the delivery system (e.g., shared vision of stakeholders), and factors related to the prevention support system (e.g., training) [21].

Similarly, a 2020 review by Herlitz et al. [19] aimed to determine if schools in high-income countries sustained public health interventions (e.g., those targeting obesity prevention, drug use, sexual health, mental health, violence, safety/accident prevention) after funding ended. This review included 24 empirical studies of 18 interventions, and mapped barriers and facilitators to three of the four main constructs of the General Theory of Implementation framework [23, 24]: capacity (i.e., staff member roles, funding and material, cognitive and social resources to sustain health interventions), potential (i.e., staff motivation and commitment), and capability (i.e., intervention adaptation and integration and wider policy context for health promotion) [19].

Both reviews provide important contributions to the field; however, they have a few notable limitations. First, although both reviews were published recently, searches were undertaken only up to 2018. Considering the rapid growth in empirical work within the field [13], it is important to capture more recent studies that may influence key conclusions and our understanding of the barriers and facilitators that impede or promote intervention sustainment. Further, the scope of study eligibility was limited within these previous reviews. For example, the review by Cassar and colleagues was limited to only include studies reporting on interventions delivered in the school setting with a primary outcome to either increase physical activity and/or decrease sedentary behaviour [21]. Studies eligible for inclusion in the review by Herlitz and colleagues did not necessarily need to report on barriers or facilitators to be included within their review, but more so focused on the sustainment or (dis)continuation of a school-based public health intervention [19]. Second, although both reviews utilised a theoretical implementation framework to synthesise their findings, neither used a specific sustainability framework that focuses on the specific issues related to intervention sustainment to contextualise their findings. Given the dynamic nature of sustainability, factors that influence the implementation of interventions may differ from those that influence intervention sustainment [25]. Thus, barriers and facilitators influencing sustainment should be assessed with a framework that focuses on longer-term sustainability. The Integrated Sustainability Framework developed by Shelton and colleagues offers a novel and promising approach for this task. Its development was informed by available empirical research on factors identified as important determinants of sustainability across a range of contexts and interventions [13]. Specifically, it aims to comprehensively identify and synthesise the dynamic interactions between multi-level factors (21 factors across five domains) that have been found to be influential to intervention sustainment across diverse types of interventions delivered in ‘real world’ clinical and community-based settings, including schools [13]. In contrast to many determinant frameworks in implementation science, this framework is specifically focused on sustainability. As such, a useful feature of this framework is that it can help identify and organise multilevel factors that may be important in facilitating sustainability, including salient outer and inner contextual factors of the organisation, process factors of intervention delivery and partnership engagement, in addition to factors related to implementer and population characteristics and characteristics of the intervention (see Table 1 for a full description of framework domains with corresponding factors included, factor definitions, and examples of application within schools and/or childcare services). Third, previous reviews have not examined the sustainment of health behaviour interventions employed in other child education settings including childcare services. Given health services and practitioners that provide support and health-promoting services to education settings, i.e., childcare and primary schools often occur by the same organisations, having an understanding of the factors that impact on the long-term delivery of interventions delivered in such settings (both similarities and differences) is important to help inform where efforts should be focused and distinctions made [26, 27]. Further, whilst there has been a plethora of implementation studies conducted in child care setting [28–30], few have examined the sustainability of these. Therefore, it is now important to determine the factors that are instrumental to the ongoing sustainment of such interventions in the childcare setting.

**Table 1:** Integrated Sustainability Framework domains with corresponding factors covered, definitions, and examples of application

Domain	Factors covered^**a**^	Factor definition^**b**^	Examples of application within schools and/or childcare services*
**Outer contextual factors**	• Policy and legislation • Sociopolitical context • Funding environment • Leadership • Values, priorities, needs • Community ownership	**Sociopolitical context:** The external landscape, including existing policies and regulations, guidelines, and mandates that pose implications on the sustainment of evidence-based interventions (EBIs). This may also include sociocontextual norms or policies that are discriminatory or stigmatising.	External attention, e.g., from government, institutions, or agencies on programmes or interventions, national certification, or government policies.
		**Funding environment and availability:** The funding landscape, including nature, stability, scope, diversity, and length of the funding environment.	Provision of funding support from external sources, e.g., government or non-governmental organisations.
		**External partnerships and leadership/environmental support:** Receiving external support through networks and partnerships (e.g., through engagement or resource exchange with academic and health organisations and community partners), support, commitment, and involvement from national leadership.	Partnership with a university or health organisation who provides support (e.g., through the provision of resources or training from a local Area Health Service).
		**Values, needs, priorities:** The extent to which an EBI or topic is regarded as a national priority or fits with national, state, or local organisational priorities, needs, and values.	Governmental policies and priorities, e.g., Federal Government prioritisation of obesity within Australia.
**Inner contextual factors**	• Funding/resources • Leadership/support • Climate/culture • Staffing turnover • Structural characteristics • Capacity • Champion • Polices (alignment) • Mission	**Programme champions:** Individuals who have strong influences on the behaviours, attitudes, and norms of their colleagues/peers and promote the ongoing delivery of EBIs.	Having an effective school champion (e.g., classroom teacher, physical education teacher, stage coordinator, or school executive) who leads and is responsible for driving the ongoing delivery of a health-promoting programme within schools.
		**Organisational leadership/support:** Support from those within the organisation who have formal responsibility for leading, organising, and overseeing the programme.	Support from school principals, executives, and other teachers.
		**Organisational readiness/resources:** The level of resources and support internally to facilitate the ongoing delivery of a programme (e.g., space, money, funding, time).	Allocated in-school funding for a health-promoting programme provided by the principal; or in-school access to resources, e.g., adequate equipment and programme materials or adequate space.
		**Organisational stability:** Staff attrition and turnover of space, organisation, staffing, or leadership.	Determining the impact of staff turnover, e.g., teaching staff, principals, and school champions on the ongoing delivery of a health-promoting programme in schools.
**Processes**	• Partnership/engagement • Training/support/supervision • Fidelity • Adaptation • Planning • Team/board functioning • Programme evaluation/data • Communication • Technical assistance • Capacity building	**Partnership/engagement:** Processes to directly and actively engage with key stakeholders (e.g., community board, role modelling, and networking).	Advisory groups and meetings with P&C committees and other stakeholders to provide updates about a health-promoting programme in schools.
		**Training/supervision/support:** Processes related to implementation strategies (e.g., formal education or training, formal supervision processes, or other forms of support).	Provision of booster workshops and training to up-skill teachers or school champions to facilitate the ongoing delivery of a health-promoting programme in schools.
		**Programme evaluation/data:** Collection of data and assessment or feedback to inform programme planning and decisions.	Conducting process evaluation surveys with participants involved, e.g., principals, teachers, parents, or students to inform what programme strategies were effective or ineffective and make improvements to facilitate the ongoing delivery of a health-promoting programme.
		**Adaptation:** Processes in place to actively and systematically guide adaptation of a policy or programme.	Implementing a plan for adaptation to enable the alteration of a health-promoting programme as required, e.g., introducing wet weather plans and plans for casual teachers. This also includes the ability to adapt a programme based on factoring including climate or geographical location, e.g., implementing a contingency plan to conduct regular physical activity within a rural school experiencing consistently hot weather.
		**Communications and strategic planning:** Processes explicitly related to or that guide the sustainment of a programme over time, e.g., through grant-writing, activities and engagement regarding sustainment, or marketing/communication plan focused on promoting the sustainment of an EBI.	Dissemination of information and promotion of a school health-promoting programme through means of school newsletters, online platforms, school social media pages, or within local newspapers.
**Characteristics of the interventionists and population**	• Provider/implementer characteristics • Implementation skills/expertise • Implementer attitudes • Implementer motivation • Population characteristics	**Implementer characteristics:** Implementer role self-efficacy, role clarity, commitment, and attitude.	Perceived personal capability, motivation, and attitudes of the teachers delivering a health-promoting programme in schools.
		**Implementer benefits and stressors:** Implementer benefits and stressors in role (including if paid or a volunteer).	Perceived personal benefit or stressors for teachers being involved in a health-promoting programme in schools, e.g., personal satisfaction knowing a programme will positively impact on students; or alternatively feeling overwhelmed with their own ability to deliver the programme given other school priorities.
		**Implementer skills/expertise:** Prior knowledge, training, and motivation of the implementer.	Perceived personal preparedness of teachers to adequately deliver the programme or intervention within schools, factoring in any previous training may have completed.
		**Population characteristics:** Trust and medical mistrust, literacy, socioeconomic status, race/ethnicity, and experiences of stigma or discrimination among the target population.	Appropriateness of the programme considering the SES of the population, e.g., rural schools. Further, this includes the appropriateness of programme resources and materials considering literacy levels of the target population.
**Characteristics of the intervention**	• Adaptability • Fit with population and context • Benefits/need • Burden/complexity • Trialability • Cost	**Adaptability of EBI/fidelity:** Degree to which an EBI can be tailored or refined to fit new settings or population needs, e.g., original guidelines or evidence vs. newer guidelines or evidence.	Adaptability of a school-based health-promoting programme for teacher’s schedule and the school environment, e.g., adaptability of a programme to include contingency plans if materials and equipment are not available.
		**Fit with context/population/organisation:** Fit of an EBI within a context, populations, and organisations as well as the perceived trust and medical mistrust of an EBI or source of evidence.	Appropriateness of a health-promoting programme considering the context, culture, and population within schools to address an identified issue, e.g., childhood obesity; and inclusion of a credible source supporting the delivery of the programme, e.g., university or Area Health Service.
		**Perceived benefits**: Perceived impact, evidence, cost, or relative advantage of an EBI.	Value of a health-promoting programme within a school. Prioritisation of the programme over other competing interests, e.g., maths and English. School staff (principal and teachers) belief that the programme will be advantageous and the cost of the programme is appropriate.
		**Perceived need**: Perceived need in the community or setting for an EBI or the topic it addresses.	The value parents see in a school health-promoting programme and the benefit this will have on their children; or regard it as a burden and prioritise other subjects over the programme. This extends to the wider school community and whether they see a need for the programme, e.g., P&C committees.

^a^An exhaustive list of factors (barriers and facilitators) for each domain regarded as particularly important across multiple settings and contexts informed by the Integrated Sustainability Framework

^b^Definitions for each factor regarded as particularly important specifically within schools and/or childcare services were informed by Shelton et al. [13] and collaboration with one of the developers of the Integrated Sustainability Framework

*Examples of how each factor could be applied within schools and/or childcare services predetermined by the research team in collaboration with one of the developers of the Integrated Sustainability Framework.

Further evidence synthesis is needed to advance the literature on sustainment of evidence-based interventions. As such, the current review seeks to address the aforementioned limitations of previous reviews. The objective of the current systematic review was to comprehensively identify and synthesise factors (barriers and facilitators) that influence the sustainment of health behaviour interventions in schools and childcare services targeting the leading risk factors for chronic disease development (poor diet, physical inactivity, obesity, tobacco smoking, and harmful alcohol use).

## Methods

### Protocol and registration

This review was prospectively registered on PROSPERO (17.1.20, CRD42020127869, [31]) and follows the Preferred Reporting Items for Systematic Reviews and Meta-Analyses (PRISMA) guidelines for reporting (see Additional file 1).

### Eligibility criteria

#### Types of articles

Articles reporting on experimental or non-experimental studies of any design that examined factors (barriers or facilitators) qualitatively and/or quantitatively related to the sustainment of a health behaviour intervention (policy, practice, or programme) in schools or childcare services were included. Articles were eligible if external support to implement the intervention had ceased at least six months prior to follow-up data collection. External support included the provision of training, funding, or resources and other implementation support by researchers or agencies tasked with implementing the health behaviour intervention.

#### Types of settings and participants

Articles of studies that conducted interventions in schools (elementary, middle, or secondary) or childcare services (pre-schools, nurseries, or long day care services) were included. Eligible participants were considered any end-user or stakeholder who may have been involved in the sustainment of an intervention in schools or childcare services. This included teachers, principals, service managers, or other relevant staff, as well as administrators or officials of other government or non-government agencies that regulate, encourage, or enforce health behaviour interventions in schools or childcare services. We did not include families and/or caregivers as participants.

#### Types of health behaviours

Articles targeting the sustainment of an intervention that addressed the following health behaviours in schools and childcare services: poor diet, physical inactivity, obesity, tobacco smoking, and harmful alcohol use were included.

#### Barriers and facilitators

Perceived factors (barriers and facilitators) that were reported to influence the sustainment of a health behaviour intervention addressing poor diet, physical inactivity, obesity, tobacco smoking, and harmful alcohol use in schools or childcare services were included. Measures of sustained behavioural effects on an individual child’s behaviour (e.g., proportion of children continuing to meet dietary guidelines following cessation of support) were not included in this review. Consistent with other reviews [32], a barrier was defined as “a circumstance or obstacle that keeps people or things apart or prevents progress” (Oxford U, n.d.), for example, lack of funding, staffing, or time. A facilitator was defined as “a person or thing that makes something possible” (Oxford U, n.d.), for example, training, availability of resources, or leadership support.

### Information sources and searches

A systematic review of eight databases (the Cochrane Central Register of Controlled trials (CENTRAL), MEDLINE, EMBASE, PsycINFO, ERIC, CINAHL, and SCOPUS) was conducted by an experienced librarian (DB) in March 2020. There was no date limit set. The search strategy (see Additional file 2) was developed based on previous reviews including filters for the following: setting (schools and childcare) [32], relevant health behaviour topics (poor diet, physical inactivity, obesity, tobacco smoking, and harmful alcohol use), and factors (barriers and facilitators) to intervention sustainment [13, 32]. Only English language articles were included in this review. The reference lists of the two aforementioned reviews that reported on the sustainment of health behaviour interventions in schools [19, 21] were hand searched, as were the reference lists of all included articles. Where necessary, corresponding authors of potentially eligible articles were contacted to clarify study details to determine eligibility.

### Article selection

Double independent screening of all titles and abstracts was conducted by pairs of review authors (AS, HB, NN, SMc) unblinded to author or journal information. Full text screening of manuscripts was also screened in duplication by four review authors (AS, HB, NN, SMc), and reasons for exclusion were recorded. Screening of articles was conducted using the Covidence systematic review software [33]. Discrepancies between reviewers regarding article eligibility were resolved by author consensus.

### Data collection process

#### Data extraction and quality assessment

Data extraction from the included articles was conducted independently by authors AS and HB. Data extraction sheets were prepared (see Additional file 3) using established recommendations for data extraction by the Cochrane Public Health Group’s guide for Developing a Cochrane Protocol [34]. Discrepancies between reviewers regarding data extraction were resolved by consensus or if required via a third reviewer (NN). The following information was extracted: intervention name, author(s), year of publication, country, setting, health behaviour(s) targeted, design, participants, sample size, participant/setting, conceptual/theoretical framework used, initial implementation support(s) offered, method(s) of data collection, time without external implementation support, and the factors (barriers and facilitators) identified regarding the sustainment of the intervention.

Quality assessment of included articles was performed independently by two authors (AS and AH) using the Mixed-Methods Appraisal Tool (MMAT) [35]. The MMAT was developed to allow the quality assessment of varying study designs through the use of a single tool consisting of different criteria for articles reporting on quantitative, qualitative, and mixed-method studies [35]. The tool includes two screening questions, in addition to five questions per study design in which response options include: ‘yes’, ‘no’, and ‘can’t tell’. The ‘can’t tell’ response category indicates the article does not report appropriate information to answer ‘yes’ or ‘no’, or that reports unclear information related to the criterion. For the purposes of this review, questions relating to the qualitative, quantitative descriptive, and mixed-method studies were included (see Additional file 4). Discrepancies between reviewers regarding quality assessment ratings were resolved by consensus.

#### Data synthesis

Barriers and facilitators reported to be important for sustainment were coded using the Integrated Sustainability Framework, a theoretically informed framework covering the unique and multidimensional factors related to longer-term intervention delivery following implementation support (i.e., sustainment) across a range of clinical and community-based settings (including schools and childcare services) [13]. This framework includes 21 groups of factors across the following five domains: outer and inner contextual factors, processes, characteristics of the interventionist/population, and characteristics of the intervention [13]. A coding manual for the Integrated Sustainability Framework was developed (see Table 1) by the authors for the purpose of this review, in collaboration with one of the developers of the framework (author RCS). This manual included the domains with corresponding factors covered, factor definitions, and examples of application within schools and/or childcare services. Double independent coding was undertaken by three review authors (AS, NN, and HB). Discrepancies between reviewers regarding coding were resolved by consensus or by a third reviewer (RCS) if required. Barriers and facilitators were presented separately for intervention sustainment within schools and childcare services. Barriers and facilitators reported qualitatively were classified by three review authors (AS, NN, and HB) into themes informed by the Integrated Sustainability Framework, with examples of qualitative participant descriptions and themes displayed in an additional file (see Additional file 5). The number of articles reporting barriers and facilitators to intervention sustainment were classified under each Integrated Sustainability Framework domain and reported descriptively as a frequency count (see Additional file 6).

## Results

### Article selection

**Fig. 1:**
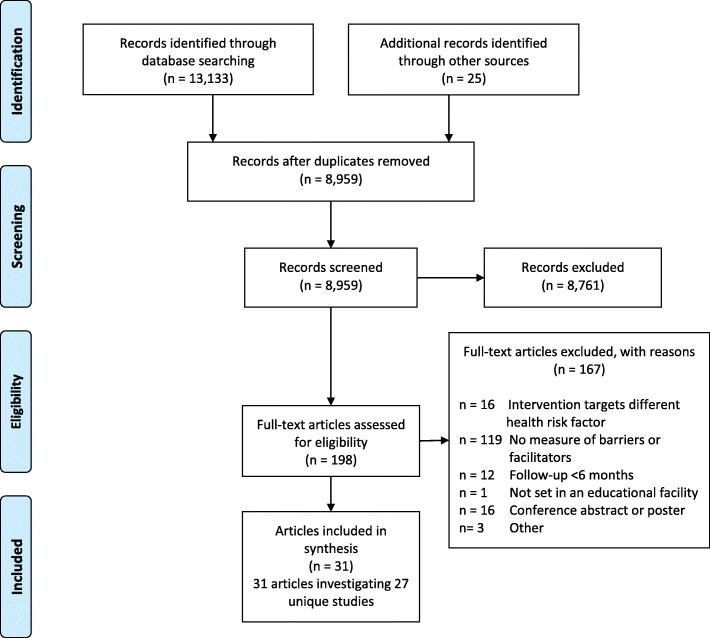
PRISMA flow diagram

The article selection and screening process is outlined in Fig. 1. Overall, 8959 unique records were screened for eligibility, of which 198 original articles were included in the full-text screen. A total of 31 articles reporting on 27 unique studies were identified for inclusion in this review [20, 36–65]. Twenty additional articles included in the current review were not examined in either recently conducted review by Cassar and colleagues [21] or Herlitz and colleagues [19]; six of which were published after mid-2018 [36, 39, 46, 49, 52, 65].

### Article characteristics

#### Type and quality of articles

The characteristics of the included articles are summarised in Table 2. Articles were published between 1993 and 2019. Studies reported in included articles were delivered in eight high-income countries, with the majority of studies conducted in the USA (*n*=19) [40, 41, 43, 44, 46, 47, 50, 52–62, 64], followed by the UK (*n*=3) [39, 42, 49], Canada (*n*=2) [20, 51], the Netherlands (*n*=2) [37, 45], Australia (*n*=2) [38, 48], Germany (*n*=1) [63], Denmark (*n*=1) [65], and Ireland (*n*=1) [36]. Twelve articles reported on studies that employed qualitative designs [36, 39, 41, 46, 47, 49, 52, 55, 60, 61, 63, 64], eight articles on studies that used quantitative designs [40, 50, 51, 53, 56–59], ten articles on studies that used mixed-method designs [20, 37, 38, 42–45, 48, 54, 65], and one was a summary article which collated previous findings and discussed lessons learned across multiple studies for specific interventions [62].

**Table 2:** Characteristics of included articles in the review

Author(s), year of publication, and ***intervention name***	Country, setting, and health behaviour(s) targeted	Design, participants/sample size, method(s) of data collection	Conceptual framework used, initial implementation support(s) offered	Time without external implementation support
Hayes et al., 2019 [36]; *Food Dudes and Green Schools Travel programmes*.	Ireland; elementary and secondary schools; diet and physical activity.	Multiple case study qualitative design; *n*=7 schools, *n*=18 participants; semi-structured interviews.	RE-AIM implementation framework [66]; - Resources/equipment - Role modelling - Incentives - Programme support officers - Programme champion/advocate.	6 months
Rozema et al., 2017 [37]; *Outdoor school ground smoking ban.*	The Netherlands; secondary schools; smoking.	Mixed-method design; *n*=15 participants; surveys and semi-structured interviews.	Conceptual sustainability framework of Shediac-Rizkallah and Bone [67]; *Initial implementation support information was unobtainable.*	6 months
Austin et al., 2011 [38]; *Promoting lifelong active youth zone programme.*	Australia; elementary schools; physical activity.	Mixed-method design; *n*=24 schools, *n*=24 participants; interviews and surveys.	RE-AIM implementation framework [66]; - Resources - Training/professional development.	6-12 months
Day et al., 2019 [39]; *PhunkyFoods nutrition and physical activity educational programme and Food Dudes Evaluation healthy eating programme*.	England; elementary schools; diet and physical activity.	Qualitative design; *n*=14 schools, *n*=64 participants; semi-structured interviews.	Durlak and DuPre implementation framework [22]; - Resources/equipment - Programme support officers - Role modelling - Incentives - Training/professional development.	6–18 months
Rohrbach et al., 1993 [40]; *Adolescent Alcohol Prevention Trial.*	USA; elementary schools; smoking and alcohol.	Quantitative 2×2 factorial design; *n*=85 participants; surveys.	Roger’s Diffusion theory [68]; - Training/professional development - Resources/equipment - Programme support officers.	1 year
Austin et al., 2006 [41]; *The School Health Index.*	USA; elementary and secondary schools; diet and physical activity.	Qualitative research design; *n*=9 schools, *n*=34 participants; in-depth interviews.	*Framework information was unobtainable*; - Programme champion/advocate.	1 year
Gorely et al., 2011 [42]; *GreatFun2Run programme.*	UK; elementary schools; diet and physical activity.	Mixed-method design; *n*=8 schools (4 intervention), 8 participants; semi-structured interviews and focus groups.	*Framework information was unobtainable*; - Resources/equipment - Announcements/marketing - Monitoring.	18–20 months
Friend et al., 2014 [43]; *‘New Moves’ physical activity programme.*	USA; secondary schools; diet and physical activity.	Mixed-method, cross-sectional design; *n*=12 schools (6 intervention), 10 participants; surveys, interviews, and direct observation.	*Framework information was unobtainable*; - Training/professional development - Resources/equipment.	1–2 years
Blaine et al., 2017 [44]; *Massachusetts Childhood Obesity Research Demonstration Intervention.*	USA; elementary and middle schools; diet and physical activity.	Convergent, parallel mixed-method design; *n*=57 participants; surveys and in-depth interviews.	Proctor et al.’s Implementation Outcomes Framework [69]; - Training/professional development - Programme champion/advocate - Resources/equipment.	1–2 years
De Meji et al., 2013 [45]; *Physical activity intervention-JUMP-in.*	The Netherlands; elementary schools; physical activity.	Mixed-method process evaluation design; *n*=9 participants; surveys and in-depth interviews.	Framework of Fleuren, Wiefferink and Paulussen [70]; - Programme support officers.	2 years
Egan et al., 2019 [46]; *Health Optimising Physical Education-based comprehensive school physical activity programme.*	USA; middle schools; physical activity.	Qualitative case study design; *n*=1 school, *n*=16 participants; semi-structured interviews and focus groups.	Roger’s Diffusion theory [68]; - Resources/equipment - Announcements/marketing - Training/professional development.	2 years
Greaney et al., 2014 [47]; *Healthy Choices Collaborative Intervention.*	USA; middle schools; diet and physical activity.	Qualitative case study design; *n*=56 participants; in-depth interviews.	*Framework information was unobtainable*; - Funding - Training/professional development - Resources/equipment.	2 years
Ward et al., 2018 [20]; *The Healthy Start-Départ Santé intervention.*	Canada; childcare services; diet and physical activity.	Mixed-method process evaluation design; *n*=140 participants; surveys (open-ended questions).	RE-AIM implementation framework [66]; - Training/professional development - Resources/equipment - Programme champion/advocate - Programme support officers.	2 years
Banfield et al., 2015 [48]; School *Youth Health Nurse programme*.	Australia; secondary schools; diet and smoking.	Mixed-method design; *n*=38 participants; programme records, surveys, and interviews.	RE-AIM implementation framework [66]; - Programme support officers - Training/professional development.	3 years
Passmore et al., 2018 [49]; *Health for Life programme.*	UK; elementary schools; diet and physical activity.	Descriptive-interpretive qualitative design; *n*=7 schools, *n*=7 participants; semi-structured interviews.	Roger’s Diffusion theory [68]; - Resources/equipment - Action plan - Training/professional development - Funding - Programme support officers.	2–5 years
Dowda et al., 2005 [50]; *Sports, Play, and Active Recreation for Kids programme.*	USA; elementary schools; physical activity.	Quantitative design; *n*=111 participants; surveys.	Roger’s Diffusion theory [68]; - Programme support officers - Training/professional development - Resources/equipment - Monitoring.	4 years
Masse et al., 2012 [51]; *Action Schools! BC physical activity and healthy eating programme.*	Canada; elementary schools; diet and physical activity.	Quantitative, cross-sectional design; *n*=720 participants; surveys.	*Framework information was unobtainable*; - Resources/equipment - Announcements/marketing - Training/professional development - Programme support officers.	4 years
Cirillo et al., 2018 [52]; *Farm to School programme—free lunches.*	USA; elementary, middle, and secondary schools; diet.	Qualitative design; *n*=10 participants; semi-structured interviews.	*Framework information was unobtainable*; - Resources/equipment - Training/professional development.	Ranged from 1–7 years (mean=4 years)
Osganian et al., 2003 [53]; *Child and Adolescent Trial for Cardiovascular Health (CATCH) programme.*	USA; elementary schools; diet.	Quantitative, cross-sectional descriptive design; *n*=272 participants; surveys.	Roger’s Diffusion theory [68]; - Resources/equipment - Programme support officers - Training/professional development.	5 years
Kelder et al., 2003 [54]; *Child and Adolescent Trial for Cardiovascular Health (CATCH) physical education (PE) programme.*	USA; elementary schools; physical activity.	Mixed-method, cross-sectional design; interviews: *n*=199 participants, questionnaires: *n*=993 participants; observation, interviews, and surveys.	*Framework information was unobtainable*; - Training/professional development - Resources/equipment.	5 years
Lytle et al., 2003 [55]; *Child and Adolescent Trial for Cardiovascular Health (CATCH) programme.*	USA; elementary schools; diet and physical activity.	Qualitative, cross-sectional design; *n*=4 schools, *n*=199 participants; interviews.	*Framework information was unobtainable*; - Training/professional development - Resources/equipment.	5 years
McKenzie et al., 2003 [56]; *Child and Adolescent Trial for Cardiovascular Health (CATCH) programme.*	USA; elementary schools; physical activity.	Quantitative, cross-sectional design; *n*=56 original intervention schools (*Participant level information was unobtainable*); surveys and direct observation.	Social Cognitive theory [71]; - Training/professional development - Resources/equipment.	5 years
Johnson et al., 2003 [57]; *Child and Adolescent Trial for Cardiovascular Health (CATCH) programme.*	USA; elementary schools; diet and physical activity.	Quantitative cross-sectional design; *n*=890 participants; surveys.	Social Cognitive theory [71]; - Training/professional development - Resources/equipment.	5 years
Hoelscher et al., 2004 [58]; *Child and Adolescent Trial for Cardiovascular Health (CATCH) programme.*	USA; elementary schools; diet and physical activity.	Quantitative, cross-sectional design; *n*=76 schools (*Participant level information was unobtainable*); surveys, direct observation.	Roger’s Diffusion theory [68]; - Training/professional development - Resources/equipment.	5 years
Parcel et al., 2003 [59]; *Child and Adolescent Trial for Cardiovascular Health (CATCH) programme.*	USA; elementary schools; diet and physical activity.	Quantitative, cross-sectional design; *n*=56 schools (*Participant level information was unobtainable*); surveys, direct observation.	Roger’s Diffusion theory [68]; - Training/professional development - Resources/equipment - Programme support officers.	5 years
Weimer et al., 2013 [60]; *Physical education programme.*	USA; elementary schools; physical activity.	Qualitative, comparative case study design; *n*=3 schools, *n*=19 participants; semi-structured interviews, direct observation, and monitoring data.	Enabling conditions framework [24]; - Programme support officers - Funding - Training/professional development - Parental/community support.	5 years
Greaney et al., 2007 [61]; *Healthy Choices Collaborative Intervention.*	USA; middle schools; diet and physical activity.	Qualitative study design/modified rapid assessment process; *n*= 21 participants; in-depth interviews.	*Framework information was unobtainable*; - Funding - Training/professional development - Resources/equipment - Programme support officers.	4–7 years
Franks et al., 2007 [62]; *Child and Adolescent Trial for Cardiovascular Health (CATCH), Planet Health and Not-On-Tobacco (N-O-T) programmes.*	USA; elementary, middle, and secondary schools; diet, physical activity and smoking.	CATCH: Mixed-method, cross-sectional design; Planet Health: Quantitative randomised controlled design; N-O-T: Matched design; *Participant level information was unobtainable*; interviews, questionnaires, and monitoring data.	Roger’s Diffusion theory [68], social cognitive theory [71]; *Initial implementation support information was unobtainable.*	4–7 years
Adametz et al., 2017 [63]; *PriMa and Torra.*	Germany; secondary schools; diet.	Descriptive-interpretive qualitative design; *n*=12 schools, *n*=13 participants; interviews.	Consolidated Framework for Implementation Research (CFIR) [72]; - Resources/equipment - Training/professional development - Programme support officers.	7–8 years
Allar et al., 2017 [64]; *Physical activity intervention- Educational resource* ‘I am Moving, I am Learning’ *resource.*	USA; childcare services; physical activity.	Qualitative design; *n*=4 teacher/staff focus groups, *n*=33 participants; focus groups.	RE-AIM implementation framework [66]; - Resources/equipment - Parental/community support.	9–10 years
Nielsen et al., 2018 [65]; *The Svendborg Project.*	Denmark; elementary schools; physical activity.	Convergent mixed-method triangulation design; *n*=6 schools, *n*=35 participants; interviews and surveys.	Stages of implementation framework [73], RE-AIM framework [66]; - Training/professional development - Programme champion/advocate.	10 years

Quality assessment ratings have been reported in an additional file (see Additional file 7). Nine of the 12 articles reporting qualitative findings rated ‘yes’ for all seven related items [36, 46, 47, 49, 55, 60, 61, 63, 64]. Comparatively, only one of the eight articles reporting quantitative descriptive findings received a ‘yes’ rating for all seven related items [50]. Notably, of all quantitative and mixed methods articles, only two articles [50, 58] received a ‘yes’ for the item ‘is the sample representative of the target population’. Further, 56% of quantitative and mixed methods articles were rated ‘no’ for the item ‘is the risk of nonresponse bias low?’ Of the ten articles reporting mixed-methods findings, no articles were rated ‘yes’ for all of the 17 related items. Reviewers found there was insufficient information to accurately rate the items ‘are divergences and inconsistencies between quantitative and qualitative results adequately addressed?’ and ‘Do the different components of the study adhere to the quality criteria of each tradition of the methods involved?’ in all articles that reported on mixed-method findings; therefore, ‘can’t tell’ ratings were deemed. Quality assessment was unable to be performed on the summary article by Franks et al. [62], as there was insufficient information on the study design to conduct a thorough appraisal of this study.

#### Types of settings and participants

Twenty-nine articles reported studies conducted in schools (17 in elementary [38–40, 42, 45, 49–51, 53–60, 65], three in middle [46, 47, 61], four in secondary [37, 43, 48, 63], two in elementary and secondary schools [36, 41], one in elementary and middle schools [44], and two in elementary, middle, and secondary schools [52, 62]) from both urban and rural areas, and of diverse high, middle, and low socioeconomic status (SES). Two articles reported studies conducted in childcare services [20, 64]. Participants of school-based articles were principals, school administrators, teachers (classroom and physical education (PE)), and teachers’ aides. Additional intervention participants in schools included programme administrators, health/food service staff, and nurses. Participants of childcare-based articles were educators and service directors/managers. The number of participants within the included articles ranged from seven to 993 (median = 36; interquartile range = 95). There were four articles that did not report outcomes at the participant level [56, 58, 59, 62]. However, for these articles, participation data was reported at the school or service level instead.

#### Types of health behaviours

The majority of articles employed interventions targeting physical activity (*n*=9) [38, 45, 46, 50, 54, 56, 60, 64, 65], followed by diet (*n*=3) [52, 53, 63], with 15 articles addressing both physical activity and diet [20, 36, 39, 41–44, 47, 49, 51, 55, 57–59, 61]. Only a small number of articles were found which focused on other health behaviours, including smoking (*n*=1) [37], smoking and alcohol (*n*=1) [40], and smoking and diet (*n*=1) [48]. One summary article addressed a combination of health behaviours, including physical activity, diet, and smoking [61].

#### Time without external implementation support

The number of years since receiving external implementation support varied, with four articles covering periods of 6 to 12 months [36–39], ten articles covering 1 to 2 years [20, 39–47], 12 articles covering 2 to 5 years [48–51, 53–60], and three with more than 5 years of follow-up [63–65]. In two articles, the number of years since receiving respective intervention implementation support ranged from 4 to 7 years [61, 62], and in one article, the time since schools received implementation support ranged from 1 to 7 years (mean = 4 years) [52].

#### Method(s) of data collection

Eight articles reported studies that used quantitative methods administered via paper-based or online questionnaires, or record audits and observations [40, 50, 51, 53, 56–59]. Thirty-nine percent (12 out of 31) of articles reported studies that used qualitative methods administered via face-to-face interviews and focus groups [36, 39, 41, 46, 47, 49, 52, 55, 60, 61, 63, 64]. A total of ten articles reported studies that used mixed-methods data collection [20, 37, 38, 42–45, 48, 54, 65], and one was a summary article which collated previous findings and discussed lessons learned across multiple studies for a specific intervention [62].

#### Conceptual/theoretical framework used

Twenty-two articles used a conceptual implementation theory/framework to guide and evaluate intervention implementation [20, 36–40, 44–46, 48–50, 53, 56–60, 62–65]. Examples of the theories/frameworks used include the following: Roger’s Diffusion theory [68]; Social Cognitive theory [71]; reach, effectiveness, adoption, implementation, and maintenance (RE-AIM) [66]; Stages of Implementation framework [73]; Consolidated Framework for Implementation Research (CFIR) [72]; Framework of Fleuren, Wiefferink and Paulussen [70]; Proctor et al.’s Implementation Outcomes Framework [69]; Enabling Conditions Framework [24]; and Durlak and DuPre’s Implementation Framework [22]. One article [37] used a sustainability-specific framework developed by Shediac-Rizkallah and Bone [67] to guide their evaluation of intervention sustainment. Nine articles did not use a conceptual framework to guide the evaluation of intervention sustainment in schools or childcare services [41–43, 47, 51, 52, 54, 55, 61].

#### Initial implementation support(s) offered

Of the 29 articles that reported on the type of initial implementation support/strategies offered, 25 reported the provision of intervention-specific resources/materials/equipment (e.g., physical activity supplies or instructional games manual) [20, 36, 38–40, 42–44, 46, 47, 49–61, 63, 64], five reported the nomination of a school champion/representative [20, 36, 41, 44, 65], 24 reported provision of skills training/professional development (e.g., teacher workshop) [20, 38–40, 43, 44, 46–61, 63, 65], four received external funding support (e.g., grant money) [47, 49, 60, 61], and 17 reported district/intervention officer support (e.g., via email/telephone correspondence or regular booster visits) [20, 36, 40, 45, 47–51, 53, 55–59, 61, 63]. All articles that reported on implementation support/strategies offered utilised at least two of the aforementioned strategies (see Table 2). Two articles did not provide information relating to the type of initial implementation support offered/received [37, 62].

### Barriers and facilitators

The most frequently identified barriers and facilitators that were reported to influence intervention sustainment are summarised below. Please see Additional file 5 for qualitative participant descriptions and Additional file 6 for a comprehensive list of identified barriers and facilitators.

#### Barriers identified as impeding intervention sustainment in schools and childcare services

Twenty-one articles (nine qualitative [36, 37, 39, 41, 46, 55, 60, 61, 63], five quantitative [40, 53, 56–58], and seven mixed-methods [42–45, 48, 54, 65]) identified 59 barriers that impeded intervention sustainment within schools which covered all five Integrated Sustainability Framework domains (see Table 3). The most frequently identified outer contextual factors as reported by principals and teachers were ‘socio-political context’ and ‘funding environment and availability’ (*n* = 6 articles respectively). Specific examples included lack of state requirements and lack of future external funding/financial support. The most frequently reported inner contextual factor and most prevalent barrier identified overall was ‘organisational readiness/resources’ (*n* = 17 articles). Specific examples included time issues/constraints, limited space/facilities, limited resources/equipment/materials, and limited internal funding. One participant description relating to this barrier was ‘We have used it, I have used it minimally I must admit, you’ve got to make time, and we did use bits and pieces that fitted in with the way that the curriculum is run in this school.’ [42]. The most frequently identified process factor was ‘training/supervision/support’ (*n* = 7 articles). A specific example included lack of training/professional development opportunities to upskill. The most frequently identified characteristic of the interventionists and population was ‘implementer characteristics’ (*n* = 8 articles). A specific example included lack of motivation/interest. Lastly, the most frequently reported characteristic of the intervention was ‘perceived benefits’ (*n* = 11 articles). Specific examples included competing resource responsibilities and curriculum demands, and low priority compared to academically oriented priorities in school.

**Table 3:** Factors (barriers and facilitators) identified to influence the sustainment of interventions in schools and childcare services

Integrated Sustainability Framework domain and factors	Barriers to sustainment of health behaviour interventions in schools and childcare (***n***=23 articles identified barriers)		Facilitators to sustainment of health behaviour interventions in schools and childcare (n=27 articles identified facilitators)	
	Schools (***n***=21 articles identified barriers)	Childcare (***n***=2 articles identified barriers)	Schools (***n***=25 articles identified facilitators)	Childcare (***n***=2 articles identified facilitators)
**Outer contextual factors**	**(*n*=13 articles)**	**(*n*=0 articles)**	**(*n*=16 articles)**	**(*n*=0 articles)**
Socio-political context	6 [36, 40, 54, 58, 60, 63]		8 [36, 37, 45, 47, 55–57, 62]	
Funding environment and availability	6 [36, 37, 39, 45, 46, 63]		6 [36, 38, 45, 47, 60, 63]	
External partnerships and leadership/environmental support	4 [36, 45, 55, 61]		9 [41–43, 45–47, 55, 62, 65]	
Values, needs and priorities	4 [36, 45, 48, 55]		4 [36, 45, 62, 65]	
**Inner contextual factors**	**(*n*=20 articles)**	**(*n*=2 articles)**	**(*n*=25 articles)**	**(*n*=0 articles)**
Programme champions	2 [44, 61]		5 [41, 44, 49, 63, 65]	
Organisational leadership/support	8 [39, 44–46, 55, 56, 61, 63]	1 [20]	22 [36–39, 41–43, 46–52, 54, 55, 57, 59, 60, 62, 63, 65]	
Organisational readiness/resources	17 [36, 37, 40–43, 46, 48, 53–58, 60, 61, 63]	1 [64]	8 [36, 37, 45, 46, 50, 56, 60, 62]	
Organisational stability	4 [43, 44, 55, 63]	1 [20]	1 [42]	
**Processes**	**(*n*=10 articles)**	**(*n*=0 articles)**	**(*n=*16 articles)**	**(*n*=0 articles)**
Partnership/engagement	3 [36, 48, 61]		6 [41, 45, 52, 60, 62, 65]	
Training/supervision/support	7 [53–57, 61, 65]		8 [39, 49, 51, 53, 55, 56, 59, 62]	
Programme evaluation/data	1 [36]		1 [65]	
Adaptation			1 [49]	
Communications and strategic planning	2 [36, 37]		6 [36, 37, 39, 49, 52, 62]	
**Characteristics of the interventionists and population**	**(*n*=11 articles)**	**(*n*=1 article)**	**(*n*=18 articles)**	**(*n=*1 article)**
Implementer characteristics	8 [36, 48, 53–55, 61, 63, 65]	1 [64]	7 [45, 47, 51, 55, 59, 63, 65]	1 [20]
Implementer benefits and stressors	2 [55, 57]	1 [64]	1 [44]	
Implementer skills/expertise	1 [57]		4 [50, 53, 55, 63]	
Population characteristics	7 [36, 37, 43, 53, 55, 61, 63]		11 [36, 37, 41, 47, 49, 54, 55, 57, 60, 62, 63]	1 [20]
**Characteristics of the intervention**	**(*n*=12 articles)**	**(*n*=1 article)**	**(*n*=15 articles)**	**(*n*=1 article)**
Adaptability of EBI/fidelity				
Fit with context/population/organisation	2 [48, 61]		4 [36, 39, 45, 49]	
Perceived benefits	11 [36, 37, 41, 42, 45, 54–56, 58, 61, 63]	1 [64]	9 [38, 43, 44, 53, 55, 56, 60, 62, 63]	1 [64]
Perceived need			3 [37, 47, 55]	

Blank cells indicate no articles reported this as a barrier or facilitator

Two articles (one qualitative [64] and one mixed-methods [20]) identified six barriers that impeded intervention sustainment within childcare services which covered three Integrated Sustainability Framework domains (see Table 3). These barriers as reported by childcare educators and managers predominantly related to ‘inner contextual factors’. Specific examples included staff resistance to change, limited space/facilities, and staff turnover. Additional identified barriers related to ‘characteristics of the interventionists and population’ included perceived duplication of tasks and staff overwhelmed with responsibilities, and ‘characteristics of the intervention’ included lack of positive programme changes in students.

#### Facilitators identified as promoting intervention sustainment in schools and childcare services

Twenty-five articles (ten qualitative [36, 39, 41, 46, 47, 49, 52, 55, 60, 63], five quantitative [50, 51, 56, 57, 59], nine mixed-methods [37, 38, 42–45, 48, 54, 65], and one summary article [62]) identified 74 facilitators that promoted intervention sustainment within schools which covered all five Integrated Sustainability Framework domains (see Table 3). The most frequently identified outer contextual factor as reported by principals and teachers was ‘socio-political context’ (*n* = 8 articles). Specific examples included ongoing national attention/political support and district support. The most frequently reported inner contextual factor and most prevalent facilitator identified overall was ‘organisational leadership or support’ (*n* = 22 articles). Specific examples included team cohesion/support/teamwork, continued administrative buy-in and support/endorsement/leadership, and programme integration or institutionalisation. Participant descriptions relating to this facilitator were ‘You have to get people on board, you have to have a good committee that’s willing to do the work, and really get out there and spread the word. But you can’t do it all yourself. I think that’s the tough part. But, I think I’m fortunate that we do have a good committee. The School Nurse is an important piece of it. The principal has to be on board. And, if they’re not, you’re not going to get very far.’ [school coordinator] [47]; and ‘You have to build it into the values of the leadership team and school so that you can’t imagine how you could ever go back.’ [participant S2] [49]. The most frequently identified process factor was ‘training/supervision/support’ (*n* = 8 articles). A specific example included training/professional development opportunities to upskill. The most frequently identified characteristic of the interventionists and population was ‘population characteristics’ (*n* = 11 articles). A specific example included community/parental support/engagement. Lastly, the most frequently reported characteristic of the intervention was ‘perceived benefits’ (*n* = 9 articles). A specific example included programme flexibility/variety.

Two articles (one qualitative [64] and one mixed-methods [20]) identified seven facilitators that promoted intervention sustainment within childcare services which covered two Integrated Sustainability Framework domains (see Table 3). These facilitators related to ‘characteristics of the interventionists and population’. Specific examples included staff motivation and community/parental support/engagement, and ‘characteristics of the intervention’ included programme flexibility/variety, cost-effectiveness/low equipment requirements, enjoyable activities adopted long-term, and ease of programme activity transitions.

## Discussion

### Summary of evidence

This review aimed to identify the barriers and facilitators that influence the sustainment of health behaviour interventions in schools and centre-based childcare services targeting the most common modifiable risk factors of chronic disease development. The review identified a number of barriers and facilitators related to intervention sustainment that mapped to all domains of the Integrated Sustainability Framework, with the most prevalent factors universally identified across schools and childcare services relating to ‘inner contextual factors’. Barriers and facilitators mapped to this domain suggest that comprehensive strategies to address these factors are required to facilitate the sustainment of such interventions in schools and childcare services, for example, executive or administrative support, staff engagement and support for the intervention, and access to relevant and essential resources or equipment.

The findings of this review were consistent with other reviews [19, 21] that have found barriers and facilitators relating to ‘inner contextual factors’ of the organisation were most prevalent within the school setting. The most frequently identified barriers and facilitators across the current and aforementioned reviews included the availability of internal funding and facilities/resources/equipment and consistent executive/administrative endorsement to support intervention institutionalisation within the organisation. Given these similarities in findings across reviews, we suggest that barriers and facilitators addressing ‘inner contextual factors’ should be of specific focus when developing sustainability strategies, as they impact on the sustainment of interventions conducted in schools. Specifically, promoting strong, continuous executive/administrative and staff engagement/support, whilst also facilitating the provision of relevant resources and programme materials/equipment, may be of benefit in supporting the sustained delivery of interventions within schools [19, 74].

Our findings, however, differed from previous reviews in that we found that numerous ‘outer contextual factors’ including external infrastructure (i.e., external socio-political landscape necessary to support intervention sustainment through existing policies and regulations, guidelines, mandates), external ongoing funding, and communication between schools and external programme organisers and/or funders (i.e., occasional email correspondence and check-ups from programme officers) were also frequently identified as influential to intervention sustainment. Whilst some external barriers and facilitators were identified in previous reviews [19, 21], we found the aforementioned barriers and facilitators were identified as of particular importance in the ongoing sustainment of intervention delivery. The discordance in findings with other reviews [19, 21] may reflect the use of different conceptual/theoretical frameworks to synthesise factors. Inconsistencies in language used across frameworks make it difficult to synthesise the research in this field and determine an accurate list of priority factors that need to be addressed, as interpretations and classification of factors may differ depending on the framework used [75]. Utilising sustainability-specific frameworks in future empirical studies of intervention continuation would ensure consistency in the language and definitions used, and that researchers examine the most important factors that influence intervention sustainment [13, 75].

Findings across school and childcare settings suggest barriers and facilitators relating to ‘inner contextual factors’ including availability of space/facilities/equipment (e.g., infrastructure), executive/leadership support present, and staff turnover/attrition were particularly important to intervention sustainment. Additionally, barriers and facilitators relating to ‘implementer characteristics’ responsible for delivering the intervention including staff motivation and ‘intervention characteristics’ such as programme flexibility and cost-effectiveness were important for intervention sustainment. Similarities in identified factors across settings suggest that future efforts to address these factors could potentially be generalised across schools and childcare services. However, to our knowledge no studies have developed strategies to support the sustainment of interventions in childcare services. Given organisational differences in setting between schools and childcare services and the notably limited research conducted in childcare services as opposed to schools in this area, it is difficult to hypothesise strategies to facilitate intervention sustainment across these settings. However based on the current evidence and theory, the followings strategies may be of particular use: (1) promoting strong, continuous executive/administrative and staff engagement/support; (2) facilitating the provision of relevant resources and programme materials/equipment, to support the sustained delivery of interventions within schools and childcare services. Given there are very few empirical studies that have assessed the effectiveness of such strategies particularly in childcare services [74], we recommend further experimental work be conducted to identify the most important factors and determine the most effective, feasible, and acceptable end-user tailored strategies to support the ongoing sustainment of evidence-based health interventions in schools and childcare settings.

### Limitations

The findings of this review should be considered in the context of its methodological limitations. First, only articles published in English were included; thus, other relevant articles arising from other non-English speaking countries may have been missed. Second, studies were undertaken in schools and childcare services from diverse geographical regions (i.e., urban and rural), with populations of varying SES and race/ethnicity; however, they were predominantly conducted in higher income countries; therefore, the extent to which these findings are generalisable to other countries is limited. Thirdly, when assessing the methodological quality of included articles, the sample representativeness of the target population and low risk of non-response bias were consistently of low quality for most included quantitative and mixed method articles, which may potentially impact on both the generalisability and validity of the results.

## Conclusions

This review comprehensively identifies and synthesises the factors related to the sustainment of health behaviour interventions in schools and centre-based childcare services using a sustainability-specific framework. Intervention sustainment in schools was predominantly dependent on their organisational readiness and resources, as well as organisational leadership and support available. These findings build on the existing literature regarding the sustainment of evidence-based interventions in schools and childcare services. Finally, these results help inform the future planning, development, and empirical testing of strategies to address barriers and facilitate the sustainment of health behaviour interventions, supporting positive, long-term health outcomes in children.

## Supplementary Information

**Additional file 1:** PRISMA guidelines for reporting.

**Additional file 2:** Search strategy and terms for each database.

**Additional file 3:** Data extraction and assessment form template.

**Additional file 4:** Quality appraisal form.

**Additional file 5:** Qualitative participant descriptions of barriers and facilitators identified.

**Additional file 6:** Comprehensive list of barriers and facilitators identified.

**Additional file 7:** Quality appraisal ratings.

## Data Availability

The completed data extraction forms for the current study are available from the corresponding author on reasonable request.

## Abbreviations

MMAT: Mixed-Methods Appraisal Tool
EBIs: Evidence-based interventions
PE: Physical education
SES: Socioeconomic status
RE-AIM: Reach, effectiveness, adoption, implementation, and maintenance
CFIR: Consolidated Framework for Implementation Research
CATCH: Child and Adolescent Trial for Cardiovascular Health
