# Supplementary material for: Barriers and facilitators influencing the sustainment of health behaviour interventions in schools and childcare services: a systematic review
Source: Implement Sci. 2021 Jun 12;16:62. doi: 10.1186/s13012-021-01134-y (PMC8199827; doi:10.1186/s13012-021-01134-y)
Supplement: Supplementary file 2 — Additional file 2: Search strategy and terms for each database. [file 13012_2021_1134_MOESM2_ESM.docx]

**Additional file 2.** Search strategy and terms for each database

| **Database** | **Records identified** | **Records after de-duplication** |
| --- | --- | --- |
| MEDLINE | 2,797 | 2,776 |
| EMBASE | 4,678 | 3,019 |
| PsycINFO | 837 | 503 |
| CINAHL | 97 | 59 |
| CENTRAL | 1,176 | 522 |
| ERIC | 1,631 | 1,197 |
| SCOPUS | 1,917 | 883 |
| **Total** | **13,133** | **8,959** |

Database: **MEDLINE**

Search Strategy:

| **#** | **Searches** | **Results** |
| --- | --- | --- |
| 1 | exp obesity/ | 204602 |
| 2 | Weight Gain/ | 30556 |
| 3 | exp Weight Loss/ | 40204 |
| 4 | obes*.mp. | 340778 |
| 5 | (weight gain or weight loss).mp. | 161423 |
| 6 | (overweight or over weight or overeat* or over eat*).mp. | 73392 |
| 7 | weight change*.mp. | 10990 |
| 8 | ((bmi or body mass index) adj2 (gain or loss or change)).mp. | 4440 |
| 9 | 1 or 2 or 3 or 4 or 5 or 6 or 7 or 8 | 474755 |
| 10 | exp Exercise/ | 186267 |
| 11 | physical inactivity.mp. | 7431 |
| 12 | physical activity.mp. | 103964 |
| 13 | Motor Activity/ | 95516 |
| 14 | (physical education or physical training).mp. [mp=title, abstract, original title, name of substance word, subject heading word, floating sub-heading word, keyword heading word, organism supplementary concept word, protocol supplementary concept word, rare disease supplementary concept word, unique identifier, synonyms] | 20155 |
| 15 | "Physical Education and Training"/ | 13316 |
| 16 | Physical Fitness/ | 26735 |
| 17 | sedentary.mp. | 32612 |
| 18 | exp Life Style/ | 89604 |
| 19 | exp Leisure Activities/ | 228463 |
| 20 | Dancing/ | 2776 |
| 21 | (exercise* adj2 aerobic*).mp. | 10078 |
| 22 | sport*.mp. | 99444 |
| 23 | ((life style or life style) adj5 activ*).mp. | 1190 |
| 24 | (dance* or dancing).mp. | 7116 |
| 25 | 10 or 11 or 12 or 13 or 14 or 15 or 16 or 17 or 18 or 19 or 20 or 21 or 22 or 23 or 24 | 582280 |
| 26 | exp Diet/ | 271279 |
| 27 | nutrition*.mp. | 372721 |
| 28 | (health* adj2 eat*).mp. | 8536 |
| 29 | Child Nutrition Sciences/ | 1133 |
| 30 | exp Fruit/ or fruit*.mp. | 173884 |
| 31 | Vegetables/ or vegetable*.mp. | 63558 |
| 32 | "Fruit and Vegetable Juices"/ | 1561 |
| 33 | canteen*.mp. | 638 |
| 34 | Food Services/ | 5466 |
| 35 | menu*.mp. | 5819 |
| 36 | (calorie or calories or kilojoule*).mp. | 25506 |
| 37 | energy density.mp. | 9584 |
| 38 | Eating/ | 51773 |
| 39 | Feeding Behavior/ or feeding behavio?r*.mp. | 85183 |
| 40 | dietary intake.mp. | 23732 |
| 41 | Food Habits/ | 79861 |
| 42 | Food/ | 32265 |
| 43 | Carbonated Beverages/ or soft drink*.mp. | 5364 |
| 44 | soda.mp. | 4195 |
| 45 | sweetened drink*.mp. | 281 |
| 46 | Dietary Fats/ | 47446 |
| 47 | confectionary.mp. | 214 |
| 48 | (school adj2 (lunch* or meal*)).mp. | 1706 |
| 49 | Menu Planning/ | 1396 |
| 50 | feeding program*.mp. | 986 |
| 51 | food program*.mp. | 572 |
| 52 | (nutrition* adj2 program*).mp. | 5982 |
| 53 | cafeteria*.mp. | 1944 |
| 54 | Nutritional Status/ | 42657 |
| 55 | 26 or 27 or 28 or 29 or 30 or 31 or 32 or 33 or 34 or 35 or 36 or 37 or 38 or 39 or 40 or 41 or 42 or 43 or 44 or 45 or 46 or 47 or 48 or 49 or 50 or 51 or 52 or 53 or 54 | 902396 |
| 56 | exp Smoking/ | 143737 |
| 57 | smoking cessation/ or "tobacco use cessation"/ | 28616 |
| 58 | Nicotine/ | 25002 |
| 59 | exp "Tobacco Use"/ or Tobacco/ | 33528 |
| 60 | ((ceas* or cess* or prevent* or stop* or quit* or abstin* or abstain* or reduc*) adj5 (smok* or tobacco or nicotine)).mp. | 69832 |
| 61 | "Tobacco Use Disorder"/ | 10939 |
| 62 | ex-smoker*.mp. | 3936 |
| 63 | anti-smoke*.mp. | 1267 |
| 64 | 56 or 57 or 58 or 59 or 60 or 61 or 62 or 63 | 220528 |
| 65 | alcohol drinking/ or binge drinking/ | 66313 |
| 66 | alcoholic intoxication/ or alcoholism/ | 84135 |
| 67 | alcohol*.mp. | 410201 |
| 68 | drink*.mp. | 185901 |
| 69 | liquor*.mp. | 10220 |
| 70 | beer*.mp. | 10811 |
| 71 | wine*.mp. | 21204 |
| 72 | spirit*.mp. | 28574 |
| 73 | drunk*.mp. | 4359 |
| 74 | intoxicat*.mp. | 55989 |
| 75 | binge*.mp. | 13963 |
| 76 | 65 or 66 or 67 or 68 or 69 or 70 or 71 or 72 or 73 or 74 or 75 | 607115 |
| 77 | 9 or 25 or 55 or 64 or 76 | 2407454 |
| 78 | barrier*.mp. | 293205 |
| 79 | (impede* or impediment*).mp. | 38101 |
| 80 | facilitat*.mp. | 506764 |
| 81 | challenge*.mp. | 617409 |
| 82 | (hindrance* or hinder*).mp. | 62512 |
| 83 | obstacle*.mp. | 46103 |
| 84 | hurdle*.mp. | 11249 |
| 85 | opportunit*.mp. | 234415 |
| 86 | adher*.mp. | 218070 |
| 87 | 78 or 79 or 80 or 81 or 82 or 83 or 84 or 85 or 86 | 1831752 |
| 88 | Child, Preschool/ | 897388 |
| 89 | (pre-school* or preschool*).mp. | 904740 |
| 90 | day care/ | 5070 |
| 91 | child care/ or childcare*.mp. | 7580 |
| 92 | (daycare* or day care*).mp. | 15097 |
| 93 | early child*.mp. | 26451 |
| 94 | nurseries.mp. or nursery/ | 4862 |
| 95 | Kinder*.mp. | 23047 |
| 96 | schools/ or schools, nursery/ | 37762 |
| 97 | ((primary or elementary or middle or junior or high or secondary) adj (school* or student*)).mp. | 64783 |
| 98 | 88 or 89 or 90 or 91 or 92 or 93 or 94 or 95 or 96 or 97 | 1027034 |
| 99 | sustain*.mp. | 332815 |
| 100 | implement*.mp. | 462685 |
| 101 | routini?ation.mp. | 215 |
| 102 | discontin*.mp. | 122765 |
| 103 | (deadopt* or "de-adopt*").mp. | 80 |
| 104 | durabil*.mp. | 19093 |
| 105 | institutionali?ation.mp. | 9151 |
| 106 | maintenance.mp. | 296820 |
| 107 | capacity building.mp. | 5759 |
| 108 | Knowledge utili?ation.mp. | 123 |
| 109 | (continual or continuous).mp. | 383695 |
| 110 | policy adherence.mp. | 39 |
| 111 | (reaim or "re aim").mp. | 554 |
| 112 | 99 or 100 or 101 or 102 or 103 or 104 or 105 or 106 or 107 or 108 or 109 or 110 or 111 | 1537767 |
| 113 | 77 and 87 and 98 and 112 | 2148 |
| 114 | limit 113 to english language | 2073 |
| **115** | **limit 114 to ed=20190530-20191213** | **122** |
| 116 | (determinant* or correlate* or mediator* or contributor* or association* or modifier* or confounder* or pattern* or relation* or predictor*).mp. | 5794036 |
| 117 | 77 and 98 and 112 and 116 | 3576 |
| 118 | limit 117 to english language | 3386 |
| **119** | **118 not 114** | **2797** |

**MEDLINE records identified n = 2,797**

**********************************************************************************

Database: **EMBASE**
Search Strategy:

| **#** | **Searches** | **Results** |
| --- | --- | --- |
| 1 | exp obesity/ | 510628 |
| 2 | weight gain/ | 91553 |
| 3 | Weight Loss.mp. or exp weight reduction/ | 149945 |
| 4 | obes*.mp. | 569410 |
| 5 | (weight gain or weight loss).mp. | 271001 |
| 6 | (overweight or over weight or overeat* or over eat*).mp. | 106411 |
| 7 | weight change*.mp. | 23186 |
| 8 | ((bmi or body mass index) adj2 (gain or loss or change)).mp. | 7644 |
| 9 | 1 or 2 or 3 or 4 or 5 or 6 or 7 or 8 | 812296 |
| 10 | exp exercise/ | 352719 |
| 11 | physical inactivity.mp. or physical inactivity/ | 12296 |
| 12 | exp physical activity/ | 416458 |
| 13 | exp motor activity/ | 557876 |
| 14 | (physical education or physical training).mp. | 23958 |
| 15 | physical education/ | 13686 |
| 16 | physical fitness.mp. or fitness/ | 43535 |
| 17 | sedentary.mp. | 44283 |
| 18 | lifestyle/ | 109573 |
| 19 | Leisure Activities.mp. or leisure/ | 36346 |
| 20 | exp sport/ | 166166 |
| 21 | dancing/ | 4800 |
| 22 | (exercise* adj2 aerobic*).mp. | 21107 |
| 23 | sport*.mp. | 147055 |
| 24 | ((lifestyle or life style) adj5 activ*).mp. | 8711 |
| 25 | (dance* or dancing).mp. | 10031 |
| 26 | 10 or 11 or 12 or 13 or 14 or 15 or 16 or 17 or 18 or 19 or 20 or 21 or 22 or 23 or 24 or 25 | 1454898 |
| 27 | exp diet/ | 358246 |
| 28 | nutrition*.mp. or nutrition/ | 523264 |
| 29 | (health* adj2 eat*).mp. | 11596 |
| 30 | Child Nutrition Sciences.mp. or nutritional science/ | 5915 |
| 31 | fruit*.mp. or fruit/ or "fruit and vegetable juice"/ | 151609 |
| 32 | vegetable*.mp. or vegetable/ | 133769 |
| 33 | canteen*.mp. | 1086 |
| 34 | Food Services.mp. or catering service/ | 19299 |
| 35 | menu.mp. | 4841 |
| 36 | (calorie or calories or kilojoule*).mp. | 45316 |
| 37 | Energy Intake.mp. or caloric intake/ | 67492 |
| 38 | energy density.mp. | 7645 |
| 39 | eating/ | 36812 |
| 40 | feeding behavio?r*.mp. or feeding behavior/ | 90017 |
| 41 | dietary intake.mp. or dietary intake/ | 91512 |
| 42 | Food Habit*.mp. | 3559 |
| 43 | food/ | 92967 |
| 44 | carbonated beverage/ or soft drink*.mp. or soft drink/ | 7676 |
| 45 | soda.mp. | 5727 |
| 46 | sweetened drink*.mp. | 392 |
| 47 | Dietary Fats.mp. or fat intake/ | 49952 |
| 48 | confectionary.mp. | 409 |
| 49 | (school adj2 (lunch* or meal*)).mp. | 2215 |
| 50 | Menu Planning.mp. | 232 |
| 51 | feeding program*.mp. | 1140 |
| 52 | food program*.mp. | 676 |
| 53 | (nutrition* adj2 program*).mp. | 7590 |
| 54 | cafeteria*.mp. | 2471 |
| 55 | nutritional status/ | 66039 |
| 56 | 27 or 28 or 29 or 30 or 31 or 32 or 33 or 34 or 35 or 36 or 37 or 38 or 39 or 40 or 41 or 42 or 43 or 44 or 45 or 46 or 47 or 48 or 49 or 50 or 51 or 52 or 53 or 54 or 55 | 1254944 |
| 57 | exp smoking/ | 386677 |
| 58 | smoking cessation/ | 57064 |
| 59 | nicotine/ | 48377 |
| 60 | "tobacco use"/ or tobacco/ | 56920 |
| 61 | ((ceas* or cess* or prevent* or stop* or quit* or abstin* or abstain* or reduc*) adj5 (smok* or tobacco or nicotine)).mp. | 94504 |
| 62 | tobacco dependence/ | 20065 |
| 63 | ex-smoker*.mp. | 7901 |
| 64 | anti-smok*.mp. | 1690 |
| 65 | 57 or 58 or 59 or 60 or 61 or 62 or 63 or 64 | 491200 |
| 66 | drinking behavior/ | 49480 |
| 67 | binge drinking/ or alcohol consumption/ | 125542 |
| 68 | alcohol intoxication/ | 14058 |
| 69 | alcoholism/ | 131005 |
| 70 | alcohol*.mp. | 684727 |
| 71 | drink*.mp. | 225055 |
| 72 | liquor*.mp. | 25970 |
| 73 | beer*.mp. | 16013 |
| 74 | wine*.mp. | 27231 |
| 75 | spirit*.mp. | 36681 |
| 76 | drunk*.mp. | 8730 |
| 77 | intoxicat*.mp. | 274344 |
| 78 | binge*.mp. | 22091 |
| 79 | 66 or 67 or 68 or 69 or 70 or 71 or 72 or 73 or 74 or 75 or 76 or 77 or 78 | 1149797 |
| 80 | 9 or 26 or 56 or 65 or 79 | 4387157 |
| 81 | barrier*.mp. | 381996 |
| 82 | (impede* or impediment*).mp. | 48333 |
| 83 | facilitat*.mp. | 636589 |
| 84 | challenge*.mp. | 781516 |
| 85 | (hindrance* or hinder*).mp. | 74911 |
| 86 | obstacle*.mp. | 58744 |
| 87 | hurdle*.mp. | 14601 |
| 88 | opportunit*.mp. | 311097 |
| 89 | adher*.mp. | 293872 |
| 90 | 81 or 82 or 83 or 84 or 85 or 86 or 87 or 88 or 89 | 2343828 |
| 91 | Child, Preschool/ | 428427 |
| 92 | (pre-school* or preschool*).mp. | 623209 |
| 93 | day care/ | 12136 |
| 94 | child care/ or childcare*.mp. | 38706 |
| 95 | (daycare* or day care*).mp. | 17066 |
| 96 | early child*.mp. | 37554 |
| 97 | nurseries.mp. or nursery/ | 6582 |
| 98 | Kinder*.mp. | 33043 |
| 99 | high school student/ or nursery school/ or school teacher/ or school child/ or middle school/ or high school/ or middle school student/ or primary school/ | 390741 |
| 100 | ((primary or elementary or middle or junior or high or secondary) adj (school* or student*)).mp. | 87742 |
| 101 | 91 or 92 or 93 or 94 or 95 or 96 or 97 or 98 or 99 or 100 | 1023128 |
| 102 | sustain*.mp. | 466886 |
| 103 | implement*.mp. | 607232 |
| 104 | routini?ation.mp. | 251 |
| 105 | discontin*.mp. | 206473 |
| 106 | (deadopt* or "de-adopt*").mp. | 77 |
| 107 | durabil*.mp. | 24200 |
| 108 | institutionali?ation.mp. | 12680 |
| 109 | maintenance.mp. | 401385 |
| 110 | capacity building.mp. | 7438 |
| 111 | Knowledge utili?ation.mp. | 152 |
| 112 | (continual or continuous).mp. | 564106 |
| 113 | policy adherence.mp. | 53 |
| 114 | (reaim or "re aim").mp. | 630 |
| 115 | 102 or 103 or 104 or 105 or 106 or 107 or 108 or 109 or 110 or 111 or 112 or 113 or 114 | 2142295 |
| 116 | 80 and 90 and 101 and 115 | 3073 |
| 117 | limit 116 to english language | 2997 |
| **118** | **limit 117 to dd=20190530-20191213** | **180** |
| 119 | (determinant* or correlate* or mediator* or contributor* or association* or modifier* or confounder* or pattern* or relation* or predictor*).mp. | 7523987 |
| 120 | 80 and 101 and 115 and 119 | 5902 |
| 121 | limit 120 to english language | 5646 |
| **122** | **121 not 117** | **4678** |

**EMBASE records identified n = 4,678**

**********************************************************************************

Database: **PsycINFO**
Search Strategy:

| **#** | **Searches** | **Results** |
| --- | --- | --- |
| 1 | Obesity/ | 23737 |
| 2 | Weight Gain/ | 3013 |
| 3 | Weight Loss/ | 3603 |
| 4 | obes*.mp. | 43226 |
| 5 | (weight gain or weight loss).mp. | 22329 |
| 6 | (overweight or over weight or overeat* or over eat*).mp. | 17673 |
| 7 | weight change*.mp. | 2177 |
| 8 | ((bmi or body mass index) adj2 (gain or loss or change)).mp. | 805 |
| 9 | 1 or 2 or 3 or 4 or 5 or 6 or 7 or 8 | 60583 |
| 10 | exp Exercise/ | 25446 |
| 11 | physical inactivity.mp. | 1942 |
| 12 | physical activity.mp. or Physical Activity/ | 36795 |
| 13 | Motor Activity.mp. | 38060 |
| 14 | (physical education or physical training).mp. | 7141 |
| 15 | Physical Education/ | 4433 |
| 16 | Physical Fitness/ | 4181 |
| 17 | sedentary.mp. | 6726 |
| 18 | exp Lifestyle/ | 11481 |
| 19 | leisure time/ or recreation/ | 10811 |
| 20 | exp Sports/ | 32513 |
| 21 | Dance/ | 2210 |
| 22 | (exercise* adj2 aerobic*).mp. | 2742 |
| 23 | sport*.mp. | 38917 |
| 24 | ((lifestyle or life style) adj5 activ*).mp. | 2421 |
| 25 | (dance* or dancing).mp. | 8356 |
| 26 | 10 or 11 or 12 or 13 or 14 or 15 or 16 or 17 or 18 or 19 or 20 or 21 or 22 or 23 or 24 or 25 | 158016 |
| 27 | Diets/ | 12467 |
| 28 | exp Nutrition/ or Nutrition*.mp. | 74781 |
| 29 | (health* adj2 eat*).mp. | 4336 |
| 30 | Child Nutrition Sciences.mp. | 192 |
| 31 | fruit*.mp. | 18055 |
| 32 | vegetable*.mp. | 5931 |
| 33 | canteen*.mp. | 135 |
| 34 | Food Services.mp. | 589 |
| 35 | menu*.mp. | 2264 |
| 36 | (calorie or calories or kilojoule*).mp. | 4534 |
| 37 | Food Intake/ or Energy Intake.mp. | 17341 |
| 38 | energy density.mp. | 313 |
| 39 | Eating.mp. | 61976 |
| 40 | Eating Behavior/ | 12458 |
| 41 | feeding behavio?r*.mp. | 21343 |
| 42 | dietary intake.mp. | 2218 |
| 43 | Food/ | 13765 |
| 44 | ((carbonated or sweetened or soft) adj (drink* or beverage*)).mp. | 1406 |
| 45 | soda.mp. | 448 |
| 46 | Dietary Fat*.mp. | 2338 |
| 47 | confectionary.mp. | 40 |
| 48 | (school adj2 (lunch* or meal*)).mp. | 666 |
| 49 | feeding program*.mp. | 124 |
| 50 | food program*.mp. | 150 |
| 51 | (nutrition* adj2 program*).mp. | 1731 |
| 52 | cafeteria*.mp. | 735 |
| 53 | 27 or 28 or 29 or 30 or 31 or 32 or 33 or 34 or 35 or 36 or 37 or 38 or 39 or 40 or 41 or 42 or 43 or 44 or 45 or 46 or 47 or 48 or 49 or 50 or 51 or 52 | 154914 |
| 54 | SMOKING CESSATION/ or exp TOBACCO SMOKING/ | 35704 |
| 55 | Nicotine/ | 10897 |
| 56 | ((ceas* or cess* or prevent* or stop* or quit* or abstin* or abstain* or reduc*) adj5 (smok* or tobacco or nicotine)).mp. | 24093 |
| 57 | ex-smoker*.mp. | 732 |
| 58 | anti-smok*.mp. | 560 |
| 59 | 54 or 55 or 56 or 57 or 58 | 43877 |
| 60 | exp Alcohol Abuse/ | 47862 |
| 61 | alcohol intoxication/ or alcohol drinking patterns/ | 24571 |
| 62 | alcohol*.mp. | 141662 |
| 63 | drink*.mp. | 64576 |
| 64 | liquor*.mp. | 981 |
| 65 | beer*.mp. | 3158 |
| 66 | wine*.mp. | 2739 |
| 67 | spirit*.mp. | 49832 |
| 68 | drunk*.mp. | 3723 |
| 69 | intoxicat*.mp. | 10482 |
| 70 | binge*.mp. | 13564 |
| 71 | 60 or 61 or 62 or 63 or 64 or 65 or 66 or 67 or 68 or 69 or 70 | 221511 |
| 72 | 9 or 26 or 53 or 59 or 71 | 540323 |
| 73 | exp PRESCHOOL STUDENTS/ or PRESCHOOL TEACHERS/ | 12541 |
| 74 | (pre-school* or preschool*).mp. | 52058 |
| 75 | day care centers/ or child day care/ | 3226 |
| 76 | (childcare* or child care*).mp. | 17839 |
| 77 | (daycare* or day care*).mp. | 8014 |
| 78 | early child*.mp. | 34528 |
| 79 | (nursery or nurseries).mp. | 5007 |
| 80 | Kindergarten Students/ or kinder*.mp. | 26132 |
| 81 | JUNIOR HIGH SCHOOLS/ or HIGH SCHOOLS/ or MIDDLE SCHOOLS/ or ELEMENTARY SCHOOLS/ | 18160 |
| 82 | ((primary or elementary or middle or junior or high or secondary) adj (school* or student*)).mp. | 191881 |
| 83 | 73 or 74 or 75 or 76 or 77 or 78 or 79 or 80 or 81 or 82 | 300095 |
| 84 | barrier*.mp. | 70084 |
| 85 | (impede* or impediment*).mp. | 13083 |
| 86 | facilitat*.mp. | 160143 |
| 87 | challenge*.mp. | 200621 |
| 88 | (hindrance* or hinder*).mp. | 16299 |
| 89 | obstacle*.mp. | 19093 |
| 90 | hurdle*.mp. | 1875 |
| 91 | opportunit*.mp. | 130799 |
| 92 | adher*.mp. | 36846 |
| 93 | 84 or 85 or 86 or 87 or 88 or 89 or 90 or 91 or 92 | 563355 |
| 94 | sustain*.mp. | 76118 |
| 95 | implement*.mp. | 170337 |
| 96 | routini?ation.mp. | 368 |
| 97 | discontin*.mp. | 20860 |
| 98 | (deadopt* or "de-adopt*").mp. | 22 |
| 99 | durabil*.mp. | 1368 |
| 100 | institutionali?ation.mp. | 8748 |
| 101 | maintenance.mp. | 61319 |
| 102 | capacity building.mp. | 2107 |
| 103 | Knowledge utili?ation.mp. | 191 |
| 104 | (continual or continuous).mp. | 55956 |
| 105 | policy adherence.mp. | 12 |
| 106 | (reaim or "re aim").mp. | 219 |
| 107 | 94 or 95 or 96 or 97 or 98 or 99 or 100 or 101 or 102 or 103 or 104 or 105 or 106 | 371265 |
| 108 | 72 and 83 and 93 and 107 | 725 |
| 109 | limit 108 to english language | 711 |
| **110** | **limit 109 to up=20190530-20191213** | **33** |
| 111 | (determinant* or correlate* or mediator* or contributor* or association* or modifier* or confounder* or pattern* or relation* or predictor*).mp. | 1699608 |
| 112 | 72 and 83 and 107 and 111 | 1145 |
| 113 | limit 112 to english language | 1099 |
| **114** | **113 not 109** | **837** |

**PsycINFO records identified n = 837**

**********************************************************************************

Database: **CINAHL**

Search Strategy:

| **#** | **Query** | **Results** |
| --- | --- | --- |
| S1 | (MH "Obesity+") | 89,816 |
| S2 | (MH "Weight Gain") | 11,134 |
| S3 | (MH "Weight Loss+") | 21,124 |
| S4 | "weight gain" or "weight loss" | 48,416 |
| S5 | overweight or "over weight" or overeat* or "over eat*" | 28,671 |
| S6 | "weight change*" | 3,649 |
| S7 | ((bmi or "body mass index") n2 (gain or loss or change)) | 3,110 |
| S8 | obes* | 120,910 |
| S9 | S1 OR S2 OR S3 OR S4 OR S5 OR S6 OR S7 OR S8 | 156,538 |
| S10 | (MH "Exercise+") | 100,369 |
| S11 | "physical inactivity" | 3,104 |
| S12 | (MH "Physical Activity") OR "physical activity" | 66,769 |
| S13 | (MH "Motor Activity+") | 11,696 |
| S14 | "physical education" or "physical training" | 5,941 |
| S15 | (MH "Physical Education and Training+") | 3,594 |
| S16 | (MH "Physical Fitness") | 15,846 |
| S17 | "sedentary" | 13,834 |
| S18 | (MH "Life Style+") | 202,604 |
| S19 | (MH "Leisure Activities+") | 62,339 |
| S20 | (MH "Sports+") | 71,113 |
| S21 | (MH "Dancing+") | 3,306 |
| S22 | exercis* n2 aerobic* | 8,670 |
| S23 | sport* | 62,768 |
| S24 | ("life style" or lifestyle) n5 activ* | 3,336 |
| S25 | dance* or dancing | 5,573 |
| S26 | S10 OR S11 OR S12 OR S13 OR S14 OR S15 OR S16 OR S17 OR S18 OR S19 OR S20 OR S21 OR S22 OR S23 OR S24 OR S25 | 451,719 |
| S27 | (MH "Diet+") | 105,849 |
| S28 | "nutrition*" | 140,366 |
| S29 | (MH "Nutrition") | 24,794 |
| S30 | health* n2 eat* | 7,016 |
| S31 | (MH "Child Nutrition") | 6,749 |
| S32 | (MH "Fruit+") | 23,195 |
| S33 | (MH "Vegetables") OR "vegetable*" | 20,531 |
| S34 | fruit* | 24,781 |
| S35 | "canteen*" | 252 |
| S36 | (MH "Food Services") | 6,347 |
| S37 | (MH "Menu Planning") OR "menu*" | 3,370 |
| S38 | calorie or calories or kilojoule* | 6,914 |
| S39 | (MH "Energy Intake") OR (MH "Food Intake") | 26,553 |
| S40 | (MH "Energy Density") OR "Energy Density" | 1,477 |
| S41 | "feeding behavio?r*" | 221 |
| S42 | (MH "Eating") OR (MH "Eating Behavior") | 19,507 |
| S43 | "dietary intake" | 7,862 |
| S44 | (MH "Food Habits") | 12,260 |
| S45 | (MH "Food") | 12,902 |
| S46 | (MH "Carbonated Beverages") OR "soft drink*" | 3,034 |
| S47 | soda | 1,122 |
| S48 | "sweetened drink*" | 178 |
| S49 | (MH "Dietary Fats") | 12,213 |
| S50 | "confectionary" OR (MH "Candy") | 631 |
| S51 | school n2 (lunch* or meal*) | 1,194 |
| S52 | "feeding program*" | 219 |
| S53 | "food program*" | 264 |
| S54 | (nutrition* n2 program*) | 3,530 |
| S55 | cafeteria* | 610 |
| S56 | (MH "Nutritional Status") | 13,498 |
| S57 | S27 OR S28 OR S29 OR S30 OR S31 OR S32 OR S33 OR S34 OR S35 OR S36 OR S37 OR S38 OR S39 OR S40 OR S41 OR S42 OR S43 OR S44 OR S45 OR S46 OR S47 OR S48 OR S49 OR S50 OR S51 OR S52 OR S53 OR S54 OR S55 OR S56 | 269,813 |
| S58 | (MH "Smoking+") | 64,835 |
| S59 | (MH "Smoking Cessation Programs") | 2,168 |
| S60 | (MH "Tobacco Abuse Control (Saba CCC)") OR (MH "Tobacco Abuse (Saba CCC)") | 2 |
| S61 | (MH "Nicotine") | 4,128 |
| S62 | (MH "Tobacco") | 7,118 |
| S63 | ((ceas* or cess* or prevent* or stop* or quit* or abstin* or abstain* or reduc*) n5 (smok* or tobacco or nicotine)) | 36,167 |
| S64 | (MH "Substance Use Disorders") | 32,678 |
| S65 | "ex-smoker*" | 966 |
| S66 | "anti-smok*" | 525 |
| S67 | S58 OR S59 OR S60 OR S61 OR S62 OR S63 OR S64 OR S65 OR S66 | 105,152 |
| S68 | (MH "Drinking Behavior") OR (MH "Alcohol Drinking") OR (MH "Binge Drinking") | 29,132 |
| S69 | (MH "Alcoholism") OR (MH "Alcohol Abuse Control (Saba CCC)") OR (MH "Alcohol Abuse (Saba CCC)") OR (MH "Alcohol Abuse") | 23,885 |
| S70 | (MH "Alcoholic Intoxication") | 3,010 |
| S71 | alcohol* | 93,640 |
| S72 | drink* | 52,291 |
| S73 | liquor* | 700 |
| S74 | beer* | 1,964 |
| S75 | wine* | 3,040 |
| S76 | spirit* | 28,253 |
| S77 | drunk* | 1,429 |
| S78 | intoxicat* | 7,404 |
| S79 | binge* | 7,073 |
| S80 | S68 OR S69 OR S70 OR S71 OR S72 OR S73 OR S74 OR S75 OR S76 OR S77 OR S78 OR S79 | 145,330 |
| S81 | barrier* | 79,959 |
| S82 | (impede* or impediment*) | 7,422 |
| S83 | facilitat* | 98,025 |
| S84 | challenge* | 163,902 |
| S85 | (hindrance* or hinder*) | 9,826 |
| S86 | obstacle* | 10,809 |
| S87 | hurdle* | 1,932 |
| S88 | opportunit* | 85,050 |
| S89 | adher* | 59,959 |
| S90 | S81 OR S82 OR S83 OR S84 OR S85 OR S86 OR S87 OR S88 OR S89 | 443,455 |
| S91 | (MH "Child, Preschool") | 188,729 |
| S92 | "pre-school*" or preschool* | 191,551 |
| S93 | (MH "Child Care") OR (MH "Child Day Care") OR (MH "Child Care (Saba CCC)") OR (MH "Child Care Providers") | 6,522 |
| S94 | childcare* or "child care*" | 9,752 |
| S95 | daycare* or "day care*" | 6,883 |
| S96 | ""early child*"" | 22,238 |
| S97 | (MH "Schools, Nursery") | 1,198 |
| S98 | nursery or nurseries | 3,541 |
| S99 | "kinder*" | 3,463 |
| S100 | (MH "Students, High School") OR (MH "Schools, Middle") OR (MH "Schools, Secondary") OR (MH "Schools, Elementary") | 16,751 |
| S101 | ((primary or elementary or middle or junior or high or secondary) n1 (school* or student*)) | 45,508 |
| S102 | S91 OR S92 OR S93 OR S94 OR S95 OR S96 OR S97 OR S98 OR S99 OR S100 OR S101 | 261,080 |
| S103 | sustain* | 66,956 |
| S104 | implement* | 177,771 |
| S105 | routinization | 143 |
| S106 | discontin* | 22,489 |
| S107 | (deadopt* or "de-adopt*") | 63 |
| S108 | durabil* | 2,546 |
| S109 | institutionalization | 4,024 |
| S110 | maintenance | 48,589 |
| S111 | "capacity building" | 2,029 |
| S112 | "Knowledge utili?ation" | 187 |
| S113 | (continual or continuous) | 59,459 |
| S114 | "policy adherence" or reaim or "re aim" | 385 |
| S115 | S103 OR S104 OR S105 OR S106 OR S107 OR S108 OR S109 OR S110 OR S111 OR S112 OR S113 OR S114 | 358,413 |
| S116 | S9 OR S26 OR S57 OR S67 OR S80 | 937,097 |
| **S117** | **S90 AND S102 AND S115 AND S116 limited to English** | **106** |
| S118 | (determinant* or correlate* or mediator* or contributor* or association* or modifier* or confounder* or pattern* or relation* or predictor*) | 61,909 |
| S119 | S102 AND S115 AND S116 AND S118 | 129 |
| **S120** | **S119 not S117 limited to English** | **97** |

**CINAHL records identified n = 97**

**************************************************************************************

Database: **CENTRAL**

Search Strategy:

| ID | Search | Results |
| --- | --- | --- |
| #1 | MeSH descriptor: [Obesity] explode all trees | 12677 |
| #2 | MeSH descriptor: [Weight Gain] this term only | 2372 |
| #3 | MeSH descriptor: [Weight Loss] explode all trees | 5810 |
| #4 | obes*:ti,ab,kw | 38900 |
| #5 | ("weight gain" or "weight loss"):ti,ab,kw | 28455 |
| #6 | (overweight or "over weight" or overeat* or "over eat*"):ti,ab,kw | 15185 |
| #7 | "weight change*":ti,ab,kw | 3102 |
| #8 | ((bmi or "body mass index") near/2 (gain or loss or change)):ti,ab,kw | 1231 |
| #9 | {or #1-#8} | 58228 |
| #10 | MeSH descriptor: [Exercise] explode all trees | 22836 |
| #11 | "physical inactivity":ti,ab,kw | 1104 |
| #12 | "physical activity":ti,ab,kw | 26788 |
| #13 | MeSH descriptor: [Motor Activity] this term only | 3722 |
| #14 | ("physical education" or "physical training"):ti,ab,kw | 3943 |
| #15 | MeSH descriptor: [Physical Education and Training] explode all trees | 1575 |
| #16 | MeSH descriptor: [Physical Fitness] this term only | 2792 |
| #17 | sedentary:ti,ab,kw | 6540 |
| #18 | MeSH descriptor: [Life Style] explode all trees | 5031 |
| #19 | MeSH descriptor: [Leisure Activities] explode all trees | 17649 |
| #20 | MeSH descriptor: [Dancing] this term only | 160 |
| #21 | (exercis* near/2 aerobic*):ti,ab,kw | 6616 |
| #22 | sport*:ti,ab,kw | 8397 |
| #23 | (("life style" or lifestyle) near/5 activ*):ti,ab,kw | 1831 |
| #24 | (dance* or dancing):ti,ab,kw | 884 |
| #25 | {or #10-#24} | 67177 |
| #26 | MeSH descriptor: [Diet] explode all trees | 17599 |
| #27 | nutrition*:ti,ab,kw | 37998 |
| #28 | (health* near/2 eat*):ti,ab,kw | 2158 |
| #29 | MeSH descriptor: [Child Nutrition Sciences] this term only | 140 |
| #30 | fruit*:ti,ab,kw | 6677 |
| #31 | MeSH descriptor: [Fruit] this term only | 1557 |
| #32 | vegetable*:ti,ab,kw | 5924 |
| #33 | MeSH descriptor: [Vegetables] this term only | 1218 |
| #34 | canteen*:ti,ab,kw | 78 |
| #35 | MeSH descriptor: [Fruit and Vegetable Juices] this term only | 209 |
| #36 | MeSH descriptor: [Food Services] this term only | 239 |
| #37 | menu*:ti,ab,kw | 2766 |
| #38 | (calorie or calories or kilojoule*):ti,ab,kw | 6219 |
| #39 | "energy density":ti,ab,kw | 585 |
| #40 | MeSH descriptor: [Eating] this term only | 2612 |
| #41 | MeSH descriptor: [Feeding Behavior] this term only | 3097 |
| #42 | "feeding behavio*":ti,ab,kw | 0 |
| #43 | "dietary intake":ti,ab,kw | 6530 |
| #44 | MeSH descriptor: [Food] this term only | 1252 |
| #45 | MeSH descriptor: [Carbonated Beverages] this term only | 164 |
| #46 | "soft drink*":ti,ab,kw | 251 |
| #47 | soda:ti,ab,kw | 381 |
| #48 | "sweetened drink*":ti,ab,kw | 24 |
| #49 | MeSH descriptor: [Dietary Fats] this term only | 3540 |
| #50 | confectionary:ti,ab,kw | 15 |
| #51 | (school near/2 (lunch* or meal*)):ti,ab,kw | 330 |
| #52 | MeSH descriptor: [Menu Planning] this term only | 39 |
| #53 | "feeding program*":ti,ab,kw | 63 |
| #54 | "food program*":ti,ab,kw | 70 |
| #55 | (nutrition* near/2 program*):ti,ab,kw | 1077 |
| #56 | cafeteria*:ti,ab,kw | 161 |
| #57 | MeSH descriptor: [Nutritional Status] this term only | 2355 |
| #58 | {or #26-#57} | 70306 |
| #59 | MeSH descriptor: [Smoke] explode all trees | 392 |
| #60 | MeSH descriptor: [Smoking Cessation] this term only | 3853 |
| #61 | MeSH descriptor: [Tobacco Use Cessation] this term only | 96 |
| #62 | MeSH descriptor: [Nicotine] explode all trees | 2414 |
| #63 | MeSH descriptor: [Tobacco Use] explode all trees | 151 |
| #64 | MeSH descriptor: [Tobacco] this term only | 158 |
| #65 | ((ceas* or cess* or prevent* or stop* or quit* or abstin* or abstain* or reduc*) near/5 (smok* or tobacco or nicotine)) | 14348 |
| #66 | MeSH descriptor: [Tobacco Use Disorder] this term only | 1529 |
| #67 | ex-smoker* | 790 |
| #68 | anti-smok* | 225 |
| #69 | {or #59-#68} | 15872 |
| #70 | MeSH descriptor: [Alcohol Drinking] this term only | 3521 |
| #71 | MeSH descriptor: [Binge Drinking] this term only | 145 |
| #72 | MeSH descriptor: [Alcoholic Intoxication] this term only | 635 |
| #73 | MeSH descriptor: [Alcohols] explode all trees | 36012 |
| #74 | alcohol* | 32011 |
| #75 | drink* | 17435 |
| #76 | liquor* | 604 |
| #77 | beer* | 2024 |
| #78 | wine* | 1556 |
| #79 | spirit* | 2580 |
| #80 | drunk* | 470 |
| #81 | intoxicat* | 3511 |
| #82 | binge* | 2148 |
| #83 | {or #70-#82} | 81069 |
| #84 | [**Error**]==> {or #9, #25, #58, #69, #83} | - |
| #85 | barrier* | 17594 |
| #86 | (impede* or impediment*) | 1792 |
| #87 | facilitat* | 28159 |
| #88 | challenge* | 28628 |
| #89 | (hindrance* or hinder*) | 2151 |
| #90 | obstacle* | 1824 |
| #91 | hurdle* | 321 |
| #92 | opportunit* | 12157 |
| #93 | adher* | 34348 |
| #94 | {or #85-#93} | 108998 |
| #95 | MeSH descriptor: [Child, Preschool] this term only | 423 |
| #96 | (pre-school* or preschool*) | 37899 |
| #97 | MeSH descriptor: [Child Care] this term only | 69 |
| #98 | childcare* | 455 |
| #99 | (daycare* or day care*) | 44371 |
| #100 | early child* | 20148 |
| #101 | MeSH descriptor: [Nurseries] this term only | 9 |
| #102 | nursery | 952 |
| #103 | kinder* | 3395 |
| #104 | MeSH descriptor: [Schools] this term only | 1831 |
| #105 | MeSH descriptor: [Schools, Nursery] this term only | 38 |
| #106 | ((primary or elementary or middle or junior or high or secondary) NEAR/1 (school* or student*)) | 8785 |
| #107 | {or #95-#106} | 103539 |
| #108 | sustain* | 39587 |
| #109 | implement* | 38840 |
| #110 | routini?ation | 10 |
| #111 | discontin* | 36377 |
| #112 | (deadopt* or "de-adopt*") | 4 |
| #113 | durabil* | 2416 |
| #114 | institutionali?ation | 831 |
| #115 | maintenance | 42078 |
| #116 | capacity building | 658 |
| #117 | Knowledge utili?ation | 2096 |
| #118 | (continual or continuous) | 56041 |
| #119 | policy adherence or reaim or "re aim" | 1518 |
| #120 | {or #108-#119} | 190028 |
| #121 | [**Error**]==> {AND #84, #94, #107, #120} with Cochrane Library publication date in The last 6 months | - |
| #122 | (determinant* or correlate* or mediator* or contributor* or association* or modifier* or confounder* or pattern* or relation* or predictor*) | 316315 |
| #123 | [**Error**]==> {AND #84, #107, #120, #122} | - |
| #124 | [**Error**]==> {NOT #123, #120} | - |
| #125 | [**Error**]==> #123 NOT #120 | - |

**CENTRAL records identified n = 1,176**

**********************************************************************************

Database: **ERIC**

Search Strategy:

ab((obes* OR "weight gain" OR "weight loss" OR overweight OR "over weight" OR overeat* OR over eat* OR "weight change*" OR ((bmi OR "body mass index") AND (gain OR loss OR change)) OR Exercis* OR "physical inactivit*" OR "physical activit*" OR "Motor Activity" OR "physical education" OR "physical training" OR "Physical Fitness" OR sedentary OR "leisure activit*" OR sport* OR dance* OR (("life style" OR lifestyle) AND activ*) OR Diet OR nutrition* OR (health* AND eat*) OR "Child Nutrition*" OR fruit* OR vegetable* OR canteen* OR menu* OR calorie OR calories OR kilojoule* OR "Energy Intake" OR "energy density" OR Eating OR "Feeding Behavio*" OR "dietary intake" OR food OR ((carbonated OR sweetened OR soft) AND (drink* OR beverage*)) OR soda OR "Dietary Fat*" OR confectionary OR (school AND (lunch* OR meal*)) OR "feeding program*" OR cafeteria* OR smok* OR nicotine OR tobacco OR "ex-smoker*" OR alcohol* OR drink* OR liquor* OR Beer* OR wine* OR spirit* OR drunk* OR intoxicat* OR binge*)) AND ab(("pre-school*" OR preschool* OR "day care*" OR daycare* OR "child care*" OR childcare* OR "early child*" OR nurseries* OR nursery OR kinder* OR school* OR student*)) AND ab((sustain* OR implement* OR routini?ation OR discontin* OR deadopt* OR "de-adopt*" OR durabil* OR institutionali?ation OR maintenance OR "capacity building" OR "knowledge utili?ation" OR continual OR continuous OR "policy adherence" OR reaim OR "re aim")) AND ab((determinant* OR correlate* OR mediator* OR contributor* OR association* OR modifier* OR confounder* OR pattern* OR relation* OR predictor*))

noft((obes* OR "weight gain" OR "weight loss" OR overweight OR "over weight" OR overeat* OR over eat* OR "weight change*" OR ((bmi OR "body mass index") AND (gain OR loss OR change)) OR Exercis* OR "physical inactivit*" OR "physical activit*" OR "Motor Activity" OR "physical education" OR "physical training" OR "Physical Fitness" OR sedentary OR "leisure activit*" OR sport* OR dance* OR (("life style" OR lifestyle) AND activ*) OR Diet OR nutrition* OR (health* AND eat*) OR "Child Nutrition*" OR fruit* OR vegetable* OR canteen* OR menu* OR calorie OR calories OR kilojoule* OR "Energy Intake" OR "energy density" OR Eating OR "Feeding Behavio*" OR "dietary intake" OR food OR ((carbonated OR sweetened OR soft) AND (drink* OR beverage*)) OR soda OR "Dietary Fat*" OR confectionary OR (school AND (lunch* OR meal*)) OR "feeding program*" OR cafeteria* OR smok* OR nicotine OR tobacco OR "ex-smoker*" OR alcohol* OR drink* OR liquor* OR Beer* OR wine* OR spirit* OR drunk* OR intoxicat* OR binge*)) AND noft(("pre-school*" OR preschool* OR "day care*" OR daycare* OR "child care*" OR childcare* OR "early child*" OR nurseries* OR nursery OR kinder* OR school* OR student*)) AND noft((sustain* OR implement* OR routini?ation OR discontin* OR deadopt* OR "de-adopt*" OR durabil* OR institutionali?ation OR maintenance OR "capacity building" OR "knowledge utili?ation" OR continual OR continuous OR "policy adherence" OR reaim OR "re aim")) AND noft((barrier* OR impede* OR impediment* OR facilitate* OR challenge* OR hindrance* OR hinder* OR obstacle* OR hurdle* OR opportunit* OR adher*))

**Issue with use of proximity operators within brackets (reverted to use of “and”)**

(determinant* or correlate* or mediator* or contributor* or association* or modifier* or confounder* or pattern* or relation* or predictor*)

**ERIC records identified n = 1,631**

**************************************************************************************

Database: **SCOPUS**

Search Strategy:

TITLE-ABS-KEY ( ( obes* OR "weight gain" OR "weight loss" OR overweight OR "over weight" OR overeat* OR over AND eat* OR "weight change*" OR ( ( bmi OR "body mass index" ) AND ( gain OR loss OR change ) ) OR exercis* OR "physical inactivit*" OR "physical activit*" OR "Motor Activity" OR "physical education" OR "physical training" OR "Physical Fitness" OR sedentary OR "leisure activit*" OR sport* OR dance* OR ( ( "life style" OR lifestyle ) AND activ* ) OR diet OR nutrition* OR ( health* AND eat* ) OR "Child Nutrition*" OR fruit* OR vegetable* OR canteen* OR menu* OR calorie OR calories OR kilojoule* OR "Energy Intake" OR "energy density" OR eating OR "Feeding Behavio*" OR "dietary intake" OR food OR ( ( carbonated OR sweetened OR soft ) AND ( drink* OR beverage* ) ) OR soda OR "Dietary Fat*" OR confectionary OR ( school AND ( lunch* OR meal* ) ) OR "feeding program*" OR cafeteria* OR smok* OR nicotine OR tobacco OR "ex-smoker*" OR alcohol* OR drink* OR liquor* OR beer* OR wine* OR spirit* OR drunk* OR intoxicat* OR binge* ) ) AND TITLE-ABS-KEY ( ( barrier* OR impede* OR impediment* OR facilitate* OR challenge* OR hindrance* OR hinder* OR obstacle* OR hurdle* OR opportunit* OR adher* ) ) AND TITLE-ABS-KEY ( ( "pre-school*" OR preschool* OR "day care*" OR daycare* OR "child care*" OR childcare* OR "early child*" OR nurseries* OR nursery OR kinder* OR school* OR student* ) ) AND TITLE-ABS-KEY ( ( sustain* OR implement* OR routini?ation OR discontin* OR deadopt* OR "de-adopt*" OR durabil* OR institutionali?ation OR maintenance OR "capacity building" OR "knowledge utili?ation" OR continual OR continuous OR "policy adherence" OR reaim OR "re aim" ) ) AND ( LIMIT-TO ( LANGUAGE , "English" ) ) AND ( LIMIT-TO ( PUBYEAR , 2020 ) OR LIMIT-TO ( PUBYEAR , 2019 ) )

**SCOPUS records identified n = 1,917**
