# Supplementary material for: Barriers and facilitators influencing the sustainment of health behaviour interventions in schools and childcare services: a systematic review
Source: Implement Sci. 2021 Jun 12;16:62. doi: 10.1186/s13012-021-01134-y (PMC8199827; doi:10.1186/s13012-021-01134-y)
Supplement: Supplementary file 3 — Additional file 3: Data extraction and assessment form template. [file 13012_2021_1134_MOESM3_ESM.docx]

**Additional file 3.** Data extraction and assessment form template

| **Study ID:** | **Report ID:** | Date form completed: |
| --- | --- | --- |
| First author: | Year of study: | Data extractor: |
| Citation: | | |

**1. General Information**

| Publication type Journal Article  Abstract  Other (specify e.g. book chapter)________________ | |
| --- | --- |
| Country of study: | |
| Funding source of study: | Potential conflict of interest from funding? Y / N / unclear |

**2. Study eligibility**

| **Study Characteristics** | | | **Page/ Para/ Figure #** |
| --- | --- | --- | --- |
| **Type of study**  (Experimental or non-experimental studies of any design that examined factors (barriers or facilitators) qualitatively and/or quantitiavely related to the sustainment of a health behaviour intervention (policy, practice or program) in schools or childcare services) |  Randomised Controlled Trial (RCT)   Cluster Randomised Controlled Trial (cluster RCT) |  Controlled Before and After (CBA) study  Contemporaneous data collection  Comparable control site  At least 2 x intervention and 2 x control clusters |  |
|  |  Interrupted Time Series (ITS)  At least 1 time point before and 1 after the intervention  Clearly defined intervention point |  Other design (specify): |  |
|  |  A process evaluation of an included study design | *Does the study design meet the criteria for inclusion?*  Yes  No 🡪**Exclude** Unclear  |  |
|  | Description in text: | |  |

| **Type of setting and participants**  (Setting: Elementary, middle, or secondary schools; or childcare services including pre-schools, nurseries, or long day care services)  Participants:  Any end-user or stakeholder who may have been involved in the sustainment of an intervention in schools or childcare services) | Describe the setting included: | | **Page/ Para/ Figure #** |
| --- | --- | --- | --- |
|  | Describe the participants included: | |  |
|  | Are participants defined as a group having specific social or cultural characteristics? | Yes  No  Unclear   Details: |  |
|  | How is the geographic boundary defined? | Details:  Specific location (e.g. state / country): |  |
|  | *Do the participants meet the criteria for inclusion?* | Yes  No 🡪**Exclude** Unclear  |  |

| **Types of health behaviours**  (Studies targeting the sustainment of an intervention that addressed the following health behaviours in schools and childcare services: poor diet, physical inactivity, obesity, tobacco smoking, and harmful alcohol use) | Type of health behaviour targeted by the intervention |  |  |
| --- | --- | --- | --- |
|  | *Does the intervention meet the criteria for inclusion?* | Yes  No  🡪**Exclude** Unclear  |  |
| **Time without external implementation support**  (eligible if external support to implement the intervention had ceased at least six months prior to follow-up data collection) | Length of time without external implementation support: | |  |
|  | *Is the duration of time without Time without external implementation support adequate for inclusion?* | Yes  No  🡪**Exclude** Unclear  |  |
| **Types of outcome measures**  (Factors that were reported to influence the sustainment of a health behaviour intervention addressing poor diet, physical inactivity, obesity, tobacco smoking, and harmful alcohol use in schools or childcare services) | List outcomes: |  |  |
|  | Outcome measured at a population level or individual level? | Details: |  |
|  | Factors (barriers and facilitators) reported to influence sustainment |  |  |
|  | *Do the outcome measures meet the criteria for inclusion?* | Yes  No  🡪**Exclude** Unclear  |  |

**Summary of assessment for inclusion**

| **Include in review**  **Exclude from review**  | |
| --- | --- |
| Independently assessed, and then compared?  Yes  No  | Differences resolved Yes  No  |
| Request further details? Yes  No  | Contact details of authors: |
| Notes: | |

**3. Study details**

| **Study intention** | **Descriptions as stated in the report/paper** | **Page/ Para/ Figure #** |
| --- | --- | --- |
| Aim of intervention | *What was the problem that this intervention was designed to address?* |  |
| Aim of study | *What was the study designed to assess? Are these clearly stated?* |  |
| Equity pointer: Social context of the study | *e.g. was study conducted in a particular setting that might target/exclude specific populations? See also Inclusion/exclusion criteria under Methods, below.* |  |
| Start and end date of the study | *Identify which elements of planning of the intervention should be included* |  |
| Location of study |  |  |
| Setting of study (e.g., school or childcare centre) |  |  |
| Design of study |  |  |
| Total study duration |  |  |

| **Methods** | **Descriptions as stated in the report/paper** | **Page/ Para/ Figure #** |
| --- | --- | --- |
| Method/s of recruitment of participants  *(How were potential participants approached and invited to participate? Where were participants recruited from? Does this differ from the intervention setting?)* |  |  |
| Inclusion/exclusion criteria for participation in study |  |  |
| Methods of data collection |  |  |
| Types of external implementation support |  |  |
| Representativeness of sample: Are participants in the study likely to be representative of the target population? |  |  |
| Statistical methods used and appropriateness of these methods | *(Check with your statistician if unsure about appropriateness)* |  |

**Results**

| **Participants**  *Include if relevant* | **Include information for each group (i.e. intervention and controls) under study** | **Page/ Para/ Figure #** |
| --- | --- | --- |
| What percentage of selected individuals agreed to participate? |  |  |
| Number and reason for (and sociodemographic differences of) withdrawals and exclusions for each intervention group |  |  |
| What percentage of participants received the allocated intervention or exposure of interest? |  |  |
| PROGRESS categories reported at baseline (indicate letters of those reported: Place of residence, race, occupation, gender, religion, education, SES, social capital) |  |  |
| Subgroups | *Enter a description of any participant subgroups from this paper to be analysed in the review.* |  |

**Outcomes**

| **Question** | **Outcome 1** | **Page/ Para/ Figure #** | **Outcome 2** | **Page/ Para/ Figure #** |
| --- | --- | --- | --- | --- |
| Is there an analytic framework applied (e.g. logic model, conceptual framework)? |  |  |  |  |
| Outcome definition (with diagnostic criteria if relevant) |  |  |  |  |
| Time points measured |  |  |  |  |
| Results relating to barriers and facilitators of sustainability |  |  |  |  |
| How is the measure applied? Telephone survey, mail survey, in person by trained assessor, routinely collected data, other |  |  |  |  |
| How is the outcome reported? Self or study assessor |  |  |  |  |
| Were PROGRESS categories analysed by outcome? Indicate the letters of those that outcomes were analysed by (place of residence, race, occupation, gender, religion, education, SES, social capital) |  |  |  |  |

**Other relevant information**

| Potential for author conflict *i.e. evidence that author or data collectors would benefit if results favoured the intervention under study or the control* |  |
| --- | --- |
| Key conclusions of the study authors |  |
| Could the inclusion of this study potentially bias the generalisability of the review? Equity pointer: Remember to consider whether disadvantaged populations may have been excluded from the study. |  |
| Is there potential for differences in relative effects between advantaged and disadvantaged populations? (e.g. are children from lower income families less likely to wear bicycle helmets) |  |
| Are interventions likely to be aimed at the disadvantaged? (e.g. school meals aimed at poor children). |  |
| Issues affecting directness  (*Note any aspects of population, intervention, etc. that affect this study’s direct applicability to the review question)* |  |
| References to other relevant studies |  |
| Additional notes by review authors |  |
| Correspondence required for further study information (from whom, what and when) |  |
