# Supplementary material for: Barriers and facilitators influencing the sustainment of health behaviour interventions in schools and childcare services: a systematic review
Source: Implement Sci. 2021 Jun 12;16:62. doi: 10.1186/s13012-021-01134-y (PMC8199827; doi:10.1186/s13012-021-01134-y)
Supplement: Supplementary file 4 — Additional file 4: Quality appraisal form. [file 13012_2021_1134_MOESM4_ESM.docx]

**Additional file 4.** Quality appraisal form (adapted from MMAT 2018)

**Qualitative articles**

Reviewer: Click here to enter text. Date: Click here to enter text.

Study author: Click here to enter text. Study year: Click here to enter text.

| Critical Appraisal questions: Qualitative studies | Response | | | Support for judgement | Location in text (pg & fig/table) |
| --- | --- | --- | --- | --- | --- |
|  | Yes | No | Can’t Tell |  |  |
| 1. Are there clear research questions? |  |  |  |  |  |
| 2. Do the collected data allow to address the research questions? |  |  |  |  |  |
| 3. Is the qualitative approach appropriate to answer the research question? |  |  |  |  |  |
| 4. Are the qualitative data collection methods adequate to address the research question? |  |  |  |  |  |
| 5. Are the findings adequately derived from the data? |  |  |  |  |  |
| 6. Is the interpretation of results sufficiently substantiated by data? |  |  |  |  |  |
| 7. Is there coherence between qualitative data sources, collection, analysis and interpretation? |  |  |  |  |  |
| Overall appraisal | Include Exclude Seek further information | | | | |
| Further comments (including reason for exclusion): | Click here to enter text. | | | | |

**Qualitative criteria**

**1. Are there clear research questions?**

**2. Do the collected data allow to address the research questions?**

**3. Is the qualitative approach appropriate to answer the research question?** The qualitative approach used in a study (see non-exhaustive list on the left side of this table) should be appropriate for the research question and problem. For example, the use of a grounded theory approach should address the development of a theory and ethnography should study human cultures and societies.

**4. Are the qualitative data collection methods adequate to address the research question?** This criterion is related to data collection method, including data sources (e.g., archives, documents), used to address the research question. To judge this criterion, consider whether the method of data collection (e.g., in depth interviews and/or group interviews, and/or observations) and the form of the data (e.g., tape recording, video material, diary, photo, and/or field notes) are adequate. Also, clear justifications are needed when data collection methods are modified during the study.

**5. Are the findings adequately derived from the data?** This criterion is related to the data analysis used. Several data analysis methods have been developed and their use depends on the research question and qualitative approach. For example, open, axial and selective coding is often associated with grounded theory, and within- and cross-case analysis is often seen in case study.

**6. Is the interpretation of results sufficiently substantiated by data?** The interpretation of results should be supported by the data collected. For example, the quotes provided to justify the themes should be adequate.

**7. Is there coherence between qualitative data sources, collection, analysis and interpretation?** There should be clear links between data sources, collection, analysis and interpretation.

**********************************************************************************

**Quantitative descriptive articles**

Reviewer: Click here to enter text. Date: Click here to enter text.

Study author: Click here to enter text. Study year: Click here to enter text.

| Critical Appraisal questions: Quantitative descriptive studies | Response | | | Support for judgement | Location in text (pg & fig/table) |
| --- | --- | --- | --- | --- | --- |
|  | Yes | No | Can’t Tell |  |  |
| 1. Are there clear research questions? |  |  |  |  |  |
| 2. Do the collected data allow to address the research questions? |  |  |  |  |  |
| 3. Is the sampling strategy relevant to address the research question? |  |  |  |  |  |
| 4. Is the sample representative of the target population? |  |  |  |  |  |
| 5. Are the measurements appropriate? |  |  |  |  |  |
| 6. Is the risk of nonresponse bias low? |  |  |  |  |  |
| 7. Is the statistical analysis appropriate to answer the research question? |  |  |  |  |  |
| Overall appraisal | Include Exclude Seek further information | | | | |
| Further comments (including reason for exclusion): | Click here to enter text. | | | | |

**Quantitative descriptive criteria**

**1. Are there clear research questions?**

**2. Do the collected data allow to address the research questions?**

**3. Is the sampling strategy relevant to address the research question?** Sampling strategy refers to the way the sample was selected. There are two main categories of sampling strategies: probability sampling (involve random selection) and non-probability sampling. Depending on the research question, probability sampling might be preferable. Nonprobability sampling does not provide equal chance of being selected. To judge this criterion, consider whether the source of sample is relevant to the target population; a clear justification of the sample frame used is provided; or the sampling procedure is adequate.

**4. Is the sample representative of the target population?** There should be a match between respondents and the target population. Indicators of representativeness include: clear description of the target population and of the sample (such as respective sizes and inclusion and exclusion criteria), reasons why certain eligible individuals chose not to participate, and any attempts to achieve a sample of participants that represents the target population.

**5. Are the measurements appropriate?** Indicators of appropriate measurements include: the variables are clearly defined and accurately measured, the measurements are justified and appropriate for answering the research question; the measurements reflect what they are supposed to measure; validated and reliability tested measures of the outcome of interest are used, variables are measured using ‘gold standard’, or questionnaires are pre-tested prior to data collection.

**6. Is the risk of nonresponse bias low?** Nonresponse bias consists of “an error of non-observation reflecting an unsuccessful attempt to obtain the desired information from an eligible unit.” (Federal Committee on Statistical Methodology, 2001, p. 6). To judge this criterion, consider whether the respondents and non-respondents are different on the variable of interest. This information might not always be reported in a paper. Some indicators of low non-response bias can be considered such as a low nonresponse rate, reasons for nonresponse (e.g., noncontacts vs. refusals), and statistical compensation for nonresponse (e.g., imputation). The nonresponse bias is might not be pertinent for case series and case report. This criterion could be adapted. For instance, complete data on the cases might be important to consider in these designs.

**7. Is the statistical analysis appropriate to answer the research question?** The statistical analyses used should be clearly stated and justified in order to judge if they are appropriate for the design and research question, and if any problems with data analysis limited the interpretation of the results.

**********************************************************************************

**Mixed method articles**

Reviewer: Click here to enter text. Date: Click here to enter text.

Study author: Click here to enter text. Study year: Click here to enter text.

| Critical Appraisal questions: Mixed method studies | Response | | | Support for judgement | Location in text (pg & fig/table) |
| --- | --- | --- | --- | --- | --- |
|  | Yes | No | Can’t Tell |  |  |
| 1. Are there clear research questions? |  |  |  |  |  |
| 2. Do the collected data allow to address the research questions? |  |  |  |  |  |
| 3. Is the qualitative approach appropriate to answer the research question? |  |  |  |  |  |
| 4. Are the qualitative data collection methods adequate to address the research question? |  |  |  |  |  |
| 5. Are the findings adequately derived from the data? |  |  |  |  |  |
| 6. Is the interpretation of results sufficiently substantiated by data? |  |  |  |  |  |
| 7. Is there coherence between qualitative data sources, collection, analysis and interpretation? |  |  |  |  |  |
| 8. Is the sampling strategy relevant to address the research question? |  |  |  |  |  |
| 9. Is the sample representative of the target population? |  |  |  |  |  |
| 10. Are the measurements appropriate? |  |  |  |  |  |
| 11. Is the risk of nonresponse bias low? |  |  |  |  |  |
| 12. Is the statistical analysis appropriate to answer the research question? |  |  |  |  |  |
| 13. Is there an adequate rationale for using a mixed methods design to address the research question? |  |  |  |  |  |
| 14. Are the different components of the study effectively integrated to answer the research question? |  |  |  |  |  |
| 15. Are the outputs of the integration of qualitative and quantitative components adequately interpreted? |  |  |  |  |  |
| 16. Are divergences and inconsistencies between quantitative and qualitative results adequately addressed? |  |  |  |  |  |
| 17. Do the different components of the study adhere to the quality criteria of each tradition of the methods involved? |  |  |  |  |  |
| Overall appraisal | Include Exclude Seek further information | | | | |
| Further comments (including reason for exclusion): | Click here to enter text. | | | | |

**Mixed method criteria**

Mixed methods (MM) research involves combining qualitative (QUAL) and quantitative (QUAN) methods. In this tool, to be considered MM, studies have to meet the following criteria (Creswell and Plano Clark, 2017): (a) at least one QUAL method and one QUAN method are combined; (b) each method is used rigorously in accordance to the generally accepted criteria in the area (or tradition) of research invoked; and (c) the combination of the methods is carried out at the minimum through a MM design (defined a priori, or emerging) and the integration of the QUAL and QUAN phases, results, and data)

**1. Are there clear research questions?**

**2. Do the collected data allow to address the research questions?**

**3. Is there an adequate rationale for using a mixed methods design to address the research question?** The reasons for conducting a mixed methods study should be clearly explained. Several reasons can be invoked such as to enhance or build upon qualitative findings with quantitative results and vice versa; to provide a comprehensive and complete understanding of a phenomenon or to develop and test instruments (Bryman, 2006).

**4. Are the different components of the study effectively integrated to answer the research question?** Integration is a core component of mixed methods research and is defined as the “explicit interrelating of the quantitative and qualitative component in a mixed methods study” (Plano Clark and Ivankova, 2015, p. 40). Look for information on how qualitative and quantitative phases, results, and data were integrated (Pluye et al., 2018). For instance, how data gathered by both research methods was brought together to form a complete picture (e.g., joint displays) and when integration occurred (e.g., during the data collection-analysis or/and during the interpretation of qualitative and quantitative results).

**5. Are the outputs of the integration of qualitative and quantitative components adequately interpreted?** This criterion is related to meta-inference, which is defined as the overall interpretations derived from integrating qualitative and quantitative findings (Teddlie and Tashakkori, 2009). Meta-inference occurs during the interpretation of the findings from the integration of the qualitative and quantitative components, and shows the added value of conducting a mixed methods study rather than having two separate studies.

**6. Are divergences and inconsistencies between quantitative and qualitative results adequately addressed?** When integrating the findings from the qualitative and quantitative components, divergences and inconsistencies (also called conflicts, contradictions, discordances, discrepancies, and dissonances) can be found. It is not sufficient to only report the divergences; they need to be explained. Different strategies to address the divergences have been suggested such as reconciliation, initiation, bracketing and exclusion (Pluye et al., 2009b). Rate this criterion ‘Yes’ if there is no divergence.

**7. Do the different components of the study adhere to the quality criteria of each tradition of the methods involved?** The quality of the qualitative and quantitative components should be individually appraised to ensure that no important threats to trustworthiness are present. To appraise 5.5, use criteria for the qualitative component (1.1 to 1.5), and the appropriate criteria for the quantitative component (2.1 to 2.5, or 3.1 to 3.5, or 4.1 to 4.5). The quality of both components should be high for the mixed methods study to be considered of good quality. The premise is that the overall quality of a mixed methods study cannot exceed the quality of its weakest component. For example, if the quantitative component is rated high quality and the qualitative component is rated low quality, the overall rating for this criterion will be of low quality.
