# Supplementary material for: Barriers and facilitators influencing the sustainment of health behaviour interventions in schools and childcare services: a systematic review
Source: Implement Sci. 2021 Jun 12;16:62. doi: 10.1186/s13012-021-01134-y (PMC8199827; doi:10.1186/s13012-021-01134-y)
Supplement: Supplementary file 5 — Additional file 5: Qualitative participant descriptions of barriers and facilitators identified. [file 13012_2021_1134_MOESM5_ESM.docx]

**Additional file 5.** Qualitative participant descriptions of identified barriers and facilitators related to intervention sustainment in schools and childcare services

| **Barriers** | **Facilitators** |
| --- | --- |
| **1. Outer contextual factors**  1.1 Sociopolitical context:  - No political endorsement for prevention  *“It would not be bad, if something like that [political endorsement of prevention effort] would exist. […] It wouldn’t have the aftertaste, that I need to defend it [in front of colleagues] since I know that it's existing anyway. Girls get eating disorders, regardless if I’m talking about this or not. It's only good if I can make them aware of it and that they can communicate about this taboo subject. That's why I would be pleased with such a possibility [political support].” [Participant (11) 3.1.3] [64]*  1.2 Funding environment and availability:  - External funding  *“I was ordering fruit (for the program), that was just too expensive to continue. I’ve tried to cut back on that, it’s like your strawberries, your blueberries, your blackberries, my manager was like you need to get your stock down.” [Catering manager, school 11, FDE] [40]*  *“Just a little bit like it turned out to be a lot of money, you know that they offered [grant funding], and then to continue that, to sustain that, has been kind of an issue, not really a big issue, but just like they offered almost an hourly rate, and the school cannot keep that up.” [Round 3 Interview] [47]*  1.3 External partnerships and leadership/environmental support:  - Transferability  *“The (UK) government had said if you don’t have a school travel plan...you will not qualify for funding for the school, so it’s very easy for a headmaster to say ...we’ll get a school travel plan together. As opposed to our program which is by invitation only, we’re trying to go in and persuade... there was no comparing like with like.” [Intermediary Agency B, Management Official] [37]*  - The need for external partnerships  *“[F]or sustainability purposes, I really think that schools need to get partnered up with a couple of significant organizations, like local fitness clubs that are willing to donate professional time, and local food establishments that can donate healthy snacks every week.” [62]*  - External CATCH university staff  *“The quality of the people working in the CATCH program was exceptional. CATCH staff did it with such enthusiasm. They made people feel special and made them feel like what they were doing was important. Every time the staff walked away from the meetings, they felt like they were doing something important.” [56]*  1.4 Values, needs, priorities:  - Dissemination  *…“We have a difficulty in getting clear data as to what is the actual full impact of the Green Schools program in a school.” [Civil Servant, Government Department B] [37]*  - Unavailability of foods via the commodity program  *“One of the reasons why CATCH failed was because the main thing is we have to sell a product. We are in here to sell the products to the kids and we are here to please the kids. Even though kids know what is good for them, they are not going to eat it. They are going to tell their other friends, “Ooo that’s nasty!” And pretty soon you got 5 or 6 kids that are standing there and saying, “Oh, I don’t want that!” . . . Sometimes the adults are worse that the kids, especially the teachers.” [56]* | **1. Outer contextual factors**  1.1 Sociopolitical context:  - Reduce MCAS related pressures  *“To focus on wellness, you have to reduce the pressure of MCAS. The way we teach, the way we structure our day, the way we use free time for students It’s all directed towards MCAS. And, until that pressure is relieved, I think districts will do the best they can, but they will not be able to do what they should do or what they could do. . You’re swimming in an uphill stream.” [School coordinator] [48]*  1.2 Funding environment and availability:  - Importance of future funding  *“…it proved much easier to sustain a budget, a net budget of XXX.” [Government Department A, Civil Servant] [37]*  *“It wouldn’t have happened without funding. There's so many things vying for those dollars and it's not just in the area of PE, it's beyond that. We look at what things we can use but then look for what other grant money there is available to value-add to what we've got.” [Principal] [39]*  *“But that funding piece is extremely important, to keep the activities going. It’s not as important in keeping, maybe, the teacher teaching the Healthy Choice piece; but, it is to get that whole activity level. And that activity level, and keeping those kids engaged… is what’s making this work. But, that takes funding.” [Food service personnel] [48]*  1.3 External partnerships and leadership/environmental support:  - Follow-up professional development support  *“Whole school training, CPD, they’ve got to come in and do that, you get these resources, you hand them out but its having the time to actually, I mean I’ve spoken to all the members of staff about it, given them the resources, but the people who actually create it, if they came in and talked to the teachers that would really help.” [43]*  - Partnerships with other agencies / organisations  *“They had perceived authority with no real partnership commitment on their side. We met monthly but whenever we had questions about funding they could never answer and had to get back to us.” [Round 1 Interview] [47]*  - Maintaining contact with outside experts  *“Just keep up with the check-ins… just someone there, kind of giving you reminders, or suggestions…‘‘Oh, did you hear about this school doing this,’’ kind of seeing what other schools are doing… tying people together.” [School coordinator] [48]*  - Research results have given the program legitimacy and political support  *“The connected research played a really important part in convincing the politicians that the Svendborg project should be a continuing part of the municipality of Svendborg ... with those positive research results it was very difficult for the municipality NOT to continue...” [School 5 - School head] [66]*  1.4 Values, needs, priorities:  - Value of program  *…“from the publication of the focused policy assessment there was a question whether we should just close the program…but I think the view from the Department would be that it is a good program.” [Government Department B, Civil Servant] [37]* |
| **2. Inner contextual factors**  2.1 Program champions:  - Program leader  *“[Y]ou have to have that team, because you have to have a leader who wants to lead it.” [62]*  2.2 Organisational leadership/support:  - Staff capacity and support  *“We have stopped the Phunky Foods club this term because they do the drama club now instead. There was only one teacher available to run it.” [Program coordinator, school 1, PFS] [40]*  *“… if we had more staff, it (cooking club) could be more of a regular thing.” [Program coordinator, school 11 FDE] [40]*  *“In schools, it takes a village, and for this [HCCI] to happen effectively, you have to have buy-in from everyone.” [62]*  - Lack of administrative support  *“Well coach, I really need to start this back up.’[He] saw the benefit, and the kids that used to come were standing around the school before and after school…and the parents, they supported it. So they found money to compensate us for our time.” (Round 3 Interviews) [47]*  *“The biggest challenge was getting the colleagues appreciation, because we needed someone to take care of the boys during that time. The appreciation of some colleagues was not really there because they had prejudices and doubted if little girls really need the prevention and that lessons would be cancelled again.” [Participant (12) 1.1.2] [64]*  2.3 Organisational readiness/resources:  - Time issues  *“We have used it, I have used it minimally I must admit, you’ve got to make time, and we did use bits and pieces that fitted in with the way that the curriculum is run in this school.” [43]*  - Effective teamwork between nurses and school staff.  *“I think it’s really important for the nurse and the principal to talk about what is and isn’t OK within that school setting.” [49]*  - Team dynamics  *“Some schools are more liberal than others…So there’s also that conversation to be held around as what are the expectations of this school and how will the nurse fit into this school, judging on the dynamics”. [School Principal] [49]*  *-* Time constraints of teachers and HCCI team members  *“I really [did not have] enough time to do the program. It just stressed me out because I’m running through the day, trying to keep up with what I do in my primary position.” [62]*  - Teamwork  *“You need the team to be able to carry the weight so that it’s not left on just one person.” [62]*  - Limited space  *“Space can also be a concern; we’re lucky here that we have the gym to be able to use it. When I was at a different school, I didn’t have the gym, so in the winter time, it was like okay I’m supposed to have them kick and throw and where can I do that?” [65]*  2.4 Organizational stability:  *(No qualitative information available)* | **2. Inner contextual factors**  2.1 Program champions:  - Program facilitator  *“Everybody in the school has a job to do, and this is in addition to their job. And having a facilitator who can lead — well, not necessarily lead, but help them lead, that can spot some of the stumbling blocks and help them get over those stumbling blocks faster, quicker, more efficiently and move towards completing action steps — is invaluable to the success of this project, to implementing the School Health Index.” [42]*  *“It’s got to go in there with an advocate, with somebody to help it, help implement that tool.” [42]*  - Health for life champion  *“An inspired cook is essential to bring about healthy changes to school dinners.” [Participant S6] [50]*  - Program champion  *“But it always needs someone who stands up for it.” [Participant (12) 3.1.2] [64]*  2.2 Organisational leadership/support:  - Team cohesion  *“The fact that the team worked as a team and included everybody else, I think really helped move this thing along quickly.” [42]*  - Continued buy-in and support from administration and staff  *“You have to get people on board, you have to have a good committee that’s willing to do the work, and really get out there and spread the word. But you can’t do it all yourself. I think that’s the tough part. But, I think I’m fortunate that we do have a good committee. The School Nurse is an important piece of it. The principal has to be onboard. And, if they’re not, you’re not going to get very far.” [School coordinator] [48]*  - Administrative support / head teacher commitment  *“… all programs are sustainable because I am interested in leading on them and passing them onto other people. I will make them sustainable at the school.” [Head teacher, school 10, FDE] [40]*  - Integration into existing structures  *...“we would have healthy eating in place for years…there was a survey done and the parents wanted a healthy eating policy.” [FD School Coordinator / Primary School Teacher] [37]*  - Leadership teamwork  *“You have to build it into the values of the leadership team and school so that you can’t imagine how you could ever go back.” [Participant S2] [50]*  - Program has to be linked to school priorities and part of the School Development Plan  *“The more you make the links the easier it becomes because you start to see them.” [Participant S5] [50]*  2.3 Organisational readiness/resources:  - Integrating the Health for Life ideology through the school ethos  *“Think really carefully about who you are as a school, your culture, values and ethos and how Health for Life will bring your values and ethos to life.” [Participant S3] [50]*  - Sustained engagement in programs and integration long-term / institutional embedment of program  *“… I think the most important thing is making it sustainable because if you have a big push to begin with and it wanes, then the impact is going to be much less. I think it’s important when we’re weaving it in, it becomes a sustainable part of what we do.” [Head teacher, school 2, PFS] [40]*  - Physical environment/location  *“This school has 205 students and 40 employees, which is an ideal situation because you don’t need cameras.” [Participant #5] [38]*  *“I’m aware of the fact that we were able to sustain the ban because of the school’s geographical location . . . the school is located in an industrial area and not in a residential area.” [Participant #3] [38]*  - Prioritization of FTS leads to embeddedness into school daily life and practice  *“We have some dedicated [FTS] staff and they are paid a nominal fee to do it. They would do it anyway because it is a passion of theirs… I give them that small stipend just to say ‘thank you’”. [53]*  2.4 Organizational stability:  *(No qualitative information available)* |
| **3. Processes**  3.1 Partnership/engagement:  *(No qualitative information available)*  3.2 Training/supervision/support:  - Lack of training/professional development opportunities to upskill  *“Our food service supervisor asked all the cooks who had been through CATCH training to go visit the schools that didn’t have training and help them get started with CATCH.” [56]*  *“I wish there were more training available from the CATCH staff. We’d take an outside source in a heartbeat. I know it is too costly to do with your program, but if you could figure out how to have a site monitor, someone checking in with us on a regular basis, I think the outcome would be beneficial.” [56]*  3.3 Program evaluation/data:  *(No qualitative information available)*  3.4 Adaptation:  *(No qualitative information available)*  3.5 Communications and Strategic Planning:  *(No qualitative information available)* | **3. Processes**  3.1 Partnership/engagement:  - The value of engaging in FTS must be experienced and communicated by a broad swath of the school community  *“When we do those activities, we see them be successful or more successful, all kids… not just the ones who struggle… it’s all kids that we see benefits for. That’s really why we’re doing it.” [53]*  - Relationships are foundational to support educational innovation and experimentation  *“The relationship that the people who are involved with food services have with kids is good and dynamic. By that I mean people know who the people are that work in the kitchen….It’s embraced in our community.” [53]*  3.2 Training/supervision/support:  - Training/professional development opportunities to upskill  *“The staff development was interesting and motivated teachers. They learned about nutrition and fitness. They got excited about it and therefore implemented it.” [56]*  *“And that made it difficult to implement in schools that hadn’t had the training. They missed a real motivational surge and missed looking at the importance and hearing from experts.” [56]*  3.3 Program evaluation/data adaptation:  -Need to pre-empt problems when starting something new, know what hurdles could be and come up with solutions  *“Sometimes the littlest things can create big hurdle—such as an outside tap for the garden.” [Participant S6] [50]*  - Need to embed all the changes within the curriculum and make sustainable links to maths, science, and RE, celebrations, topics and themes  *“Cooking and growing can be part of creative curriculum everywhere.” [Participant S5] [50]*  3.4 Adaptation:  *(No qualitative information available)*  3.5 Communications and Strategic Planning:  - Communication about programs  *“Communication within staff talking about things really (is important for sustaining programs).” [Year 4 teacher, school 3, PFS] [40]* |
| **4. Characteristics of the interventionists and population**  4.1 Implementer Characteristics:  - Student confidentiality  *“There’s a lot of pressure in the schools to provide information about students. They have a different level of confidentiality. So it’s really working out that common good, for what’s best for the student … as well as providing confidential service, because we won’t see anybody if it’s not confidential.” [School Youth Health Nurse] [49]*  - Duplication of tasks  *“Sometimes it feels like it’s a duplication of what’s being done with [gold] for like traveling and balance things that are already assessed in the [goal/gold] assessment, so it’s sort of a duplication for the Choosy assessment that we do.” [65]*  4.2 Implementer benefits and stressors:  - Overwhelmed with responsibilities  *“I think sometimes time can get in the way of being able to do conferences and home visits, getting your assessment done, then getting IMIL done all on top of each other can sometimes be a lot.” [65]*  - Frustration of trying to offer healthy foods at school when parents continue to serve their children less healthful foods at home  *“We are preparing one meal a day, and the parents are feeding them the other two. That child is going to go home and eat something totally to offset what we’ve just done. It doesn’t make a bit of sense to us.” [56]*  4.3 Implementer Skills/Expertise:  *(No qualitative information available)*  4.4 Population characteristics:  - Establishing parental buy-in  *“Parental buy-in—that’s how you keep it sustained after they leave here.” [62]* | **4. Characteristics of the interventionists and population**  4.1 Implementer Characteristics:  - Teachers found program helpful with transitions from one activity to the next  *“For transitions and things too cause sometimes I will use the hoop to have kids go through to line up. And taking turns going through the spatial awareness hoop to get to line up or jumping over a line as they go to give them something to focus on or do on the way.” [65]*  - Intervention delivery  *“I think both are good. One teacher needs more freedom in delivering the intervention and the other wants fully-prepared sessions with small potential for changes but less preparation effort […].” [Participant (10) 5.2.4] [64]*  4.2 Implementer benefits and stressors:  *(No qualitative information available)*  4.3 Implementer Skills/Expertise:  *(No qualitative information available)*  4.4 Population characteristics:  - Family support  *“I think a lot of it is home life, if the parents don’t push them towards sporting activities then you’re fighting a battle straight away in school.” [43]*  - Social environment (peers and parents)  *“Smoking parents don’t think it’s dangerous for their child to smoke. Parents say: ‘I allow my child to smoke at home, so I don’t understand why it’s forbidden to smoke at school’ I consider this very problematic and prefer to have consensus about this.” [38]*  - Engaging parents & developing the pupil voice to have a say on the themes of the program  *“Engaging parents is really important but don’t be dependent on parents to get things started or to continue things as it makes it very difficult to remain sustainable as there are too many factors out of your control.” [Participant S3] [50]* |
| **5. Characteristics of the intervention**  5.1 Adaptability of EBI/fidelity:  *(No qualitative information available)*  5.2 Fit with context/population/organisation:  - Ensuring effective and timely integration into new schools  *“Some schools are more liberal than others…So there’s also that conversation to be held around as what are the expectations of this school and how will the nurse fit into this school, judging on the dynamics”. [School Principal] [49]*  - Maintaining the focus on health promotion  *“Well I consider what I do actually is health promotion… I suppose they mean more as a whole school health promotion. So for example, we had a number of students that were diabetics and so … I got them together and they formed a bit of a group and we did a thing at assembly on what is diabetes, how do you look after a friend with diabetes and stuff like that, so that kind of whole school thing. But I think every day I do health promotion in the stuff that I do with young people.” [School Youth Health Nurse] [49]*  - Adopting a new curriculum  *“[W]e’ve always been encouraged to use curricula as resource, but to build our own programs. So, I would be kind of hesitant if you’re required to use... just one canned program.” [62]*  5.3 Perceived benefits:  - Program/policy conflicting with student academic achievement  *“Well, unfortunately . . . right now their priority is the [state standardized testing] and the No Child Will Be Left Behind. . . and unfortunately, maybe as a result, other issues are left behind.” [42]*  - Competing resources and curriculum demands  *“To be honest we didn’t use the resource pack for the planning base, we’ve got lots of different resources, but we went mainly from the objectives in the QCA [Qualification and Curriculum Authority] units”. [43]*  - Side effects and lack of enforcement  *“The second thing we noticed is that when you prohibit students smoking on school grounds, they search for a place to smoke outside school grounds and they become very vulnerable to those who want to sell drugs to our students. Thus, students have become a very easy target for drug dealers.” [Participant #13] [38]*  - Progression of students  *“Some of the returners are already at 3’s on everything, so next year I’m like well I’m going to assess them again, but they’re going to be a 3 already, so they have nowhere to progress to.” [65]*  - Curriculum focus  *“After all, this plan, this rigid fixed plan [curriculum] inhibits. […] You need to put your main focus on major subjects, which are relevant to the exams.” [Participant (2) 2.1.1] [64]*  - Other more academically oriented priorities in school  *“I think all the academic focus coming from the state has really made it so that if you’re going to prioritize, you’re going to prioritize on academics. . . . You always concentrate on academics but there was more room for PE and health and those kinds of things before the state kicked in the really extremely rigorous academic standards.” [56]*  5.4 Perceived need:  *(No qualitative information available)* | **5. Characteristics of the intervention**  5.1 Adaptability of EBI/fidelity:  *(No qualitative information available)*  5.2 Fit with context/population/organisation:  - Sustained engagement in programs  *“We need to reflect on these things at different part of the year, make sure we have the skills in, people share what’s worked well.” [Head teacher, school 4, PFS] [40]*  5.3 Perceived benefits:  - Students have unique roles within the classroom  *“Choosy is good role model in school and they need same at home which this makes me want to follow through at home also.” [65]*  - Intervention benefit  *"The good thing about this is in playing these games you learn social skills; obviously hopscotch, you have to take turns; skipping, someone has to hold the rope; and they're incidentally learning all the time, and the other thing is that you're taking kids away from being in the situation where they're going to have antisocial behavior; they're having success and they're happy.” [Principal] [39]*  - Flexibility  *“I think most of the classrooms are really good about just incorporating it within their day rather than saying okay now it’s time for IMIL. I think everyone just kind of like does it like as their large group or just kind of does it when it works for them.” [65]*  - Cost-effectiveness/low equipment requirements  *“Cause it’s not like you have to go out and buy that Lakeshore kit for 500-and-some dollars per classroom to implement… you don’t necessarily need a whole lot of stuff.” [65]*  - Enjoyable  *“Our song selector, whoever the song selector is they always seem to choose a Choosy song, and then everybody follows whatever the song selector does choose.” [65]*  - Program materials  *“The material was prepared professionally. It was varied, since you had the choice between worksheets, film sequences and posters.” [Participant (12) 5.2.3] [64]*  - The curriculum contained interesting, fun activities that both students and teachers enjoyed and the curriculum was organized and presented in a teacher-friendly manner  *“I love the teacher’s manual. It provides you with such a good script. If I choose to read it verbatim, literally, I could do that if I wanted to. That’s nice in this day and age because there a lot of materials out there that really do not give you enough support.” [56]*  - CATCH curriculum provided a good link to the family  *“Kids look forward to it . . . they love to go home and make the snacks together. There are some foods that they’ve never tried. We hear, “This is good, I’m going to go home and make it” so it brings the families together that way.” [56]*    5.4 Perceived need:  - Necessity of parental and community support  *“Some community support… some more grants to maybe, keep the thing going. And you know, you can do, find people that would be willing to donate their time, rather than pay to like run after school programs; and really hit the parents, and see who’s got some talents out there.” [School coordinator] [48]* |

Note: These reflect examples of qualitative descriptions of barriers and facilitators related to intervention sustainment only and do not represent an exhaustive list of identified factors.
