# Supplementary material for: Barriers and facilitators influencing the sustainment of health behaviour interventions in schools and childcare services: a systematic review
Source: Implement Sci. 2021 Jun 12;16:62. doi: 10.1186/s13012-021-01134-y (PMC8199827; doi:10.1186/s13012-021-01134-y)
Supplement: Supplementary file 6 — Additional file 6: Comprehensive list of barriers and facilitators identified. [file 13012_2021_1134_MOESM6_ESM.docx]

**Additional file 6.** Comprehensive list of identified barriers and facilitators related to intervention sustainment in schools and childcare services

| **Barriers** | **Facilitators** |
| --- | --- |
| **1. Outer contextual factors**  1.1 Sociopolitical context  - No political/district endorsement for intervention [41, 64]  - Lack of infrastructure [37]  - Lack of state requirements [55, 59, 61]  1.2 Funding environment and availability  - Lack of future external funding/financial support [37, 38, 40, 46, 47, 64]  1.3 External partnerships and leadership/environmental support  - Transferability [37]  - Insufficient communication with between schools and program organisers and/or funders [37]  - Need for external partnerships [62]  - External CATCH university staff [56]  - Complexity of external multidisciplinary collaboration [46]  1.4 Values, needs, priorities  - Dissemination [37]  - Negative external peer pressure [37]  - Government perceptions [37]  - Unavailability of foods via the commodity program [56]  - Lack of evidence-based treatments [46]  - Government stakeholders expressed that expansion of the program beyond the pilot presented challenges [49] | **1. Outer contextual factors**  1.1 Sociopolitical context  - Intervention reduced MCAS related pressures [48]  - Ongoing national attention/political support [37, 46]  - District support [56-58]  - Legislation [38]  - State educational standards [63]  1.2 Funding environment and availability  - Future external funding/financial support [37, 39, 46, 48, 61, 64]  1.3 External partnerships and leadership/environmental support  - Follow-up professional development support [43]  - Maintaining contact and partnerships with other agencies/organisations/experts [47, 48, 63]  - Continued participation of all external partners [46]  - External facilitator support [42, 56]  - Research results have given the program legitimacy and political support [66]  - Receiving teacher training on the program from the research team [44]  1.4 Values, needs, priorities  - Political goodwill/value of intervention for ongoing use [37, 46, 66]  - National implementation of the pupil follow-up system [46]  - Widespread dissemination of the intervention [46]  - Scientific evidence supporting ongoing program delivery [63] |
| **2. Inner contextual factors**  2.1 Program champions  - Program leader/facilitator/champion [45, 62]  2.2 Organisational leadership/support  - Lack of administrative buy-in and support/leadership/management [45-47, 56, 57, 62, 64]  - Staff capacity and support [40]  - Lack of centralised coordination [46]  - Resistance to change [21]  - Team cohesion/support/teamwork [56]  2.3 Organisational readiness/resources  - Time issues/constraints [37, 41, 43, 44, 54-56, 58, 59, 61, 62, 64]  - Team dynamics/attitudes [49, 62, 64]  - Limited space/facilities (e.g., classroom size) [37, 38, 44, 56, 57, 61, 65]  - Limited resources/equipment/materials [38, 42, 54-59, 64]  - Limited internal funding [42, 44, 47, 54, 56, 58, 61, 62, 64]  2.4 Organisational stability  - Staff turnover [21, 44, 45, 56, 64] | **2. Inner contextual factors**  2.1 Program champions  - Program leader/facilitator/champion [42, 45, 50, 64, 66]  2.2 Organisational leadership/support  - Team cohesion/support/teamwork [42, 49, 50, 53, 55, 56, 58, 61, 63]  - Continued administrative buy-in and support/endorsement/leadership [39, 40, 43, 44, 48, 50, 51, 55, 56, 58, 60, 61, 63, 64]  - Program linked to school priorities [50]  - Staff involvement [38, 47, 63]  - Program integration/institutionalisation [37, 40, 50, 52, 53, 60, 66]  2.3 Organisational readiness/resources  - Physical environment/location [38]  - School culture [38]  - Whole school policy [37, 38]  - Internal funding [46, 47, 61]  - Adequate equipment/resources/materials [51, 63]  - Adequate time [57]  2.4 Organisational stability  - Staff turnover [43] |
| **3. Processes**  3.1 Partnership/engagement  - Partnership engagement securing funding for program [37]  - Lack of effective collaboration between nurses and school staff [49]  - Lack of collaboration with community groups [62]  3.2 Training/supervision/support  - Lack of training/professional development opportunities to upskill [54-58, 62, 66]  3.3 Program evaluation/data  - Accuracy in assessing impact of program [37]  - Lack of clear data on effectiveness of program [37]  3.4 Adaptation  N/A  3.5 Communications and Strategic Planning  - Translation of a research-based intervention into a national program [37]  - Absence of plan with defined measures [37]  - Communicating information to stakeholders [38] | **3. Processes**  3.1 Partnership/engagement  - The value of program engagement must be experienced and communicated by a broad swath of the school community [53]  - Relationships and collaboration are foundational to support educational innovation and experimentation [53]  - Collaboration with external facilitators [42, 61, 63, 66]  - Formalisation of collaborations [46]  3.2 Training/supervision/support  - Training/professional development opportunities to upskill [40, 50, 52, 54, 56, 57, 60, 63]  3.3 Program evaluation/data  - Research results have given the program legitimacy [66]  3.4 Adaptation  - Need to pre-empt problems when starting something new and know what hurdles could be to come up with solutions [50]  3.5 Communications and Strategic Planning  - Communication of benefits/value of program [38, 40, 50, 53, 63]  - Dissemination of program information [37, 63] |
| **4. Characteristics of the interventionists and population**  4.1 Implementer Characteristics  - Student confidentiality [49]  - Perceived duplication of tasks [65]  - Lack of motivation/interest [37, 54-56, 64]  - Lack of enjoyment [62]  - Poor knowledge/training [56, 66]  4.2 Implementer benefits and stressors  - Overwhelmed with responsibilities [65]  - Frustration of trying to offer healthy foods at school when parents continue to serve their children less healthful foods at home [56]  4.3 Implementer Skills/Expertise  - Lack of prior educational requirements [58]  4.4 Population characteristics  - Social environment (e.g., peers and parents) [38]  - Lack of parental buy-in/support (e.g., uninterested parents) [37, 56, 62, 64]  - Low levels of motivation among students [44]  - Student preference [54] | **4. Characteristics of the interventionists and population**  4.1 Implementer Characteristics  - Intervention delivery [64]  - Realistic outcome expectations about tasks and responsibilities [46, 52, 60]  - Self-efficacy [60]  - Staff enjoyment [21, 56]  - Perceived importance of participation and program goals [21, 46, 66]  - Staff motivation [21, 48]  - Needs of staff [49]  4.2 Implementer benefits and stressors  - Intervention involvement acknowledged [45]  4.3 Implementer Skills/Expertise  - Prior knowledge/training/experience [51, 54, 56, 64]  - Prior educational level [54]  4.4 Population characteristics  - Social environment (e.g., peers and parents) [38]  - Education level of students [38]  - Community/parental support/engagement [21, 37, 42, 48, 50, 55, 56, 58, 61, 63, 64]  - Developing the pupil voice to have a say on the themes of the program [50] |
| **5. Characteristics of the intervention**  5.1 Adaptability of EBI/fidelity  N/A  5.2 Fit with context/population/organisation  - Ensuring effective and timely integration into new schools [49]  - Maintaining the focus on health promotion [49]  - Adopting a new curriculum [62]  5.3 Perceived benefits  - Time required to implement and record uptake of the program [37]  - Side effects and lack of enforcement [38]  - Competing resources responsibilities and curriculum demands [37, 43, 46, 56, 62, 64]  - Lack of positive program change for students [65]  - Low priority compared to academically oriented priorities in school [42, 55-57, 59]  - Need for improved match of tasks with regular functions and available skills [46]  - Cost-effectiveness/feasibility of program [56]  - Lack of program flexibility/engagement/structure [62]  5.4 Perceived need  N/A | **5. Characteristics of the intervention**  5.1 Adaptability of EBI/fidelity  N/A  5.2 Fit with context/population/organisation  - Sustained engagement in programs in organisation [40]  - Development of an internal database [37]  - Need for increased effective smart planning and controlled action [46]  - Need for compact and simplified methods, instruments, protocols and tasks [46]  - Need for additional program components (e.g., aimed at healthy eating) [46]  - Clear recommendations for an improved fit and reallocation of tasks [46]  - Need to embed all the changes within the curriculum and make sustainable links to mathematics, science, and RE, celebrations, topics and themes [50]  5.3 Perceived benefits  - Intervention benefit [39]  - Students have unique roles within the classroom [65]  - Program flexibility/variety [39, 61, 63, 64, 65]  - Cost-effectiveness/low equipment requirements [64, 65]  - Program materials [64]  - The curriculum contained interesting, fun activities that both students and teachers enjoyed and the curriculum was organized and presented in a teacher-friendly manner [56]  - Enjoyable activities adopted long-term [45, 65]  - CATCH curriculum provided a good link to the family [56]  - Teachers believing in the importance and benefits of program [44, 56]  - Program kept practitioner-friendly [63]  - Greater utilisation and structural implementation and quality control of program components/materials/resources [54, 57, 63, 64]  - Formation of habit [56]  - Helpful with transitions between program activities [65]  5.4 Perceived need  - Necessity of parental and community support [48, 56]  - Guidelines applying to everyone [38] |
